# Cyanopeptolins with Trypsin and Chymotrypsin Inhibitory Activity from the Cyanobacterium *Nostoc edaphicum* CCNP1411

**DOI:** 10.3390/md16070220

**Published:** 2018-06-26

**Authors:** Hanna Mazur-Marzec, Anna Fidor, Marta Cegłowska, Ewa Wieczerzak, Magdalena Kropidłowska, Marie Goua, Jenny Macaskill, Christine Edwards

**Affiliations:** 1 Division of Marine Biotechnology, Faculty of Oceanography and Geography, University of Gdańsk, Marszałka J. Piłsudskiego 46, PL-81378 Gdynia, Poland; anna.fidor77@gmail.com; 2 Institute of Oceanology, Polish Academy of Sciences, Powstańców Warszawy 55, PL-81712 Sopot, Poland; mceglowska@iopan.pl; 3 Department of Biomedical Chemistry, Faculty of Chemistry, University of Gdańsk, Wita Stwosza 63, PL-80308 Gdańsk, Poland; ewa.wieczerzak@ug.edu.pl (E.W.); magdalena.kropidlowska@phdstud.ug.edu.pl (M.K.); 4 School of Pharmacy and Life Sciences, Robert Gordon University, Aberdeen AB10 7GJ, UK; m.goua@rgu.ac.uk (M.G.); j.s.macaskill@rgu.ac.uk (J.M.); c.edwards@rgu.ac.uk (C.E.)

**Keywords:** cyanobacteria, *Nostoc*, cyanopeptolins, protease inhibitors

## Abstract

Cyanopeptolins (CPs) are one of the most frequently occurring cyanobacterial peptides, many of which are inhibitors of serine proteases. Some CP variants are also acutely toxic to aquatic organisms, especially small crustaceans. In this study, thirteen CPs, including twelve new variants, were detected in the cyanobacterium *Nostoc edaphicum* CCNP1411 isolated from the Gulf of Gdańsk (southern Baltic Sea). Structural elucidation was performed by tandem mass spectrometry with verification by NMR for CP962 and CP985. Trypsin and chymotrypsin inhibition assays confirmed the significance of the residue adjacent to 3-amino-6-hydroxy-2-piperidone (Ahp) for the activity of the peptides. Arginine-containing CPs (CPs-Arg^2^) inhibited trypsin at low IC_50_ values (0.24–0.26 µM) and showed mild activity against chymotrypsin (IC_50_ 3.1–3.8 µM), while tyrosine-containing CPs (CPs-Tyr^2^) were selectively and potently active against chymotrypsin (IC_50_ 0.26 µM). No degradation of the peptides was observed during the enzyme assays. Neither of the CPs were active against thrombin, elastase or protein phosphatase 1. Two CPs (CP962 and CP985) had no cytotoxic effects on MCF-7 breast cancer cells. Strong and selective activity of the new cyanopeptolin variants makes them potential candidates for the development of drugs against metabolic disorders and other diseases.

## 1. Introduction

**Figure 1:**
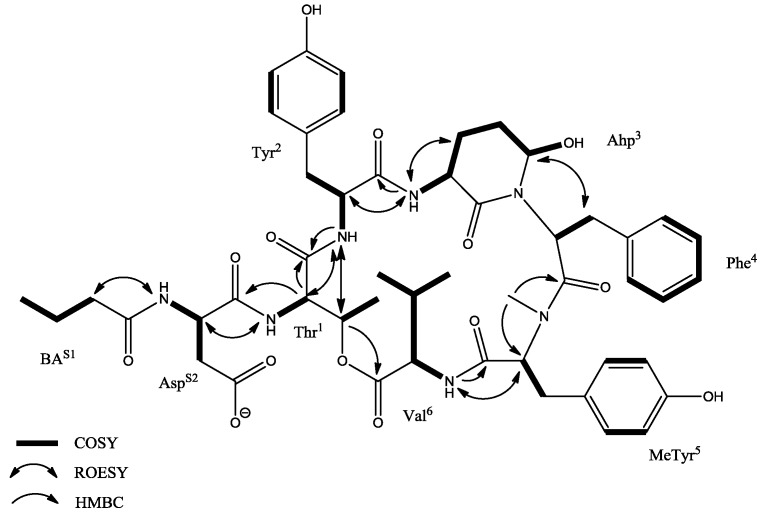
COSY, ROESY, and HMBC correlations in cyanopeptolin CP985.

**Figure 2:**
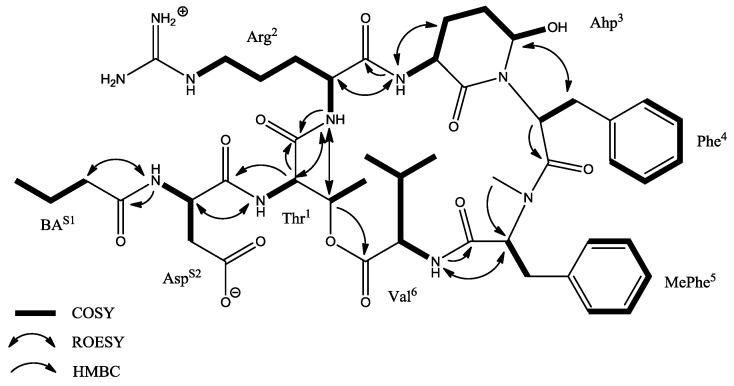
Key COSY, ROESY, and HMBC correlations in cyanopeptolin CP962.

Cyanobacterial peptides belong to the most interesting group of natural bioactive products. Initially, they were recognized as hepatotoxic and inflammatory agents. In the late 1970s, the therapeutic potential of the compounds attracted the attention of the scientific community [1,2]. Since then, numerous and structurally diverse cyanopeptides have been identified, including potent anticancer agents such as dolastatin 10, cryptophycin 52, largazol, and apratoxin [3,4,5,6,7]. Cyanopeptides have also been recognized as potent inhibitors of key metabolic enzymes, targeting mainly serine proteases and protein phosphatases [7,8,9]. Among these compounds, cyanopeptolins (CPs), a large family of cyclic depsipeptides (peptidolactones), are commonly produced by different cyanobacterial genera, including *Microcystis*, *Planktothrix*, *Anabaena* and *Nostoc*. These nonribosomal peptides (NRPs) are composed of a six-amino acid ring and a side chain with one or two residues. All CPs are characterized by the presence of 3-amino-6-hydroxy-2-piperidone (Ahp) in position 3 (Figure 1 and Figure 2). In a few CP-type peptides, the occurrence of *O*-methylated Ahp (Amp) in this position has been reported [10,11,12]. Ahp is also present in other cyclodepsipeptides such as aeruginopeptins, micropeptins, microcystilide, nostopeptins, and oscillapeptins. Position 1 in CP-type peptides is conserved and occupied by l-threonine with β-hydroxy group linked by an ester bond to carboxy terminus of amino acid in position 6 (i.e., Val, Ile or *allo*-Ile). Position 2 is most variable and occupied by residues differing in structure and polarity (e.g., Arg, Leu, Gln, Tyr, Phe, MeTyr, H_4_Tyr, Dhb) [13]. In position 4, Leu/Ile, Phe or Thr can be found, while in position 5, *N*-methylated aromatic amino acids, *N*-MeTyr or MePhe, or their homo-variants are usually present. In some CPs, Tyr or *N*-MeTyr were modified by chlorination [14]. The side chain is attached via the amino group on Thr. Usually, two major types of side chains were reported: with 1–2 amino acid residues (e.g., Asp, Glu) and aliphatic fatty acid of variable length or with glyceric acid directly linked to Thr or to an amino acid side chain [15,16,17]. Glyceric acid can be modified by sulfation or/and *O*-methylation [11,18]. In the side chain of aeruginopeptins, micropeptins or microcystilide, a hydroxyphenyl lactic acid (Hpla) was reported [19,20,21].

Cyanopeptolins, like other nonribosomal peptides, are synthesized on large multi-enzyme complexes with modular structure. The general organization of the gene clusters encoding the enzymes in different cyanobacteria is similar, however, some differences in the specificity of adenylation domains and in the presence of tailoring domains exist [22,23,24]. In the CP gene cluster from *Microcystis* and *Anabaena*, a halogenase gene was present [23,24], whereas in *Planktothrix* a glyceric acid-activating domain and sulfotransferase domain occurred [22]. The modifications in gene clusters and differences in substrate specificity of adenylation domains result in intra- and interspecies diversity of CP structures.

The majority of CPs showed inhibitory activity against serine proteases, such as trypsin, chymotrypsin, thrombin, and elastase (e.g., [12,25,26,27,28,29,30]). Cyanopeptolins with one (CP S) or two sulphate groups (CP SS) also inhibited plasmin [18]. The activity of the peptides was found to be determined by the residue in position 2, however, the significance of other structural elements was also reported [12]. In ichthyopeptins, CP analogues with 2-hydroxy-3-(4′-hydroxyphenyl)lactic acid (PAA) in the side chain, strong antiviral activity against influenza A virus was observed [31]. Tests on small crustaceans revealed the harmful effects of Ahp-containing cyclic depsipeptides [16,32,33]. For CP SS, the toxicity against *Daphnia magna* was even higher than for microcystin-LR [10], the most widely studied cyanobacterial toxin.

In cyanobacterial strains from the *Nostoc* genus, typical CP variants produced by *Microcystis* have not been reported. However, several other CP-type structures, namely nostopeptins, insulapeptolides, and nostocyclins were identified (Table 1) [34,35,36,37,38]. Nostopeptin A and B from *N. minutum* NIES-26, with 3-hydroxy-4-methylproline (Hmp) in position 1, showed inhibitory activity against elastase and chymotrypsin, but were inactive against papain, trypsin, thrombin, and plasmin [35]. Insulapeptolides A–D from *N. insulare* are characterized by the presence of Hmp in position 1 and citrulline (Cit) in the side chain. Extracts containing these peptides potently and selectively inhibited human leukocyte elastase (HLE) [38]. Nostocyclin from *Nostoc* sp. DUN901 has d-Hpla in the side chain and two homoserine residues (Hse): one in a ring part and one in a side chain of the molecule [34]. The peptide was not toxic in mouse bioassay, but showed weak activity against protein phosphatases [34,39].

Among cyanobacterial strains from the same species, significant differences in the peptide profiles are frequently reported. In our study, the structures of CPs produced by *N. edaphicum* CCNP1411 isolated from coastal waters of the Gulf of Gdańsk, southern Baltic Sea, were elucidated. In total, thirteen CP variants were identified. They represent structures typical of CPs from *Microcystis*, but different from CP-type peptides previously found in other *Nostoc* strains. The biological activity of the peptides against serine proteases, protein phosphatase 1, and MCF-7 breast cancer cells were assessed. 

## 2. Results

### 2.1. LC-MS/MS Analysis of Cyanopeptolins

**Figure 3:**
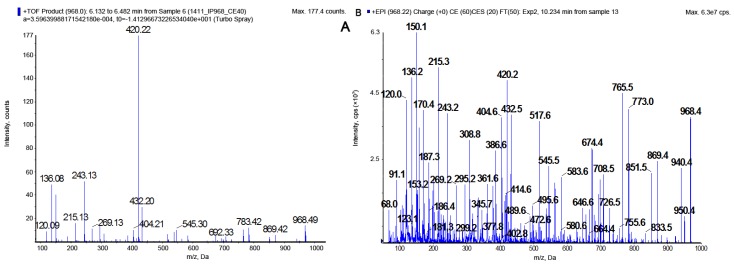
The product ion mass spectra of CP985 [Thr+Tyr+Ahp+Phe+MeTyr+Va]Asp+BA with precursor ion [M + H − H_2_O] at *m*/*z* 968. The spectra were recorded with application of a hybride quadrupole/time-of-flight mass spectrometer (QTOF) (**A**) and a hybride triple quadrupole/linear ion trap mass spectrometer (QTRAP) (**B**). The mass signals were assigned to the following fragments: 950 [M + H − 2H_2_O]^+^, 869 [M + H − Val − H_2_O]^+^, 851 [M + H − Val − 2H_2_O]^+^, 773 [M + 2H − (BA+Asp) − CO]^+^, 765 [M + 2H − (BA + Asp) − 2H_2_O]^+^, 692 [M + H − (Val + MeTyr) − H_2_O]^+^, 674 [M+H−(Val+MeTyr)−2H_2_O]^+^, 646 [M + H − (Val + MeTyr) − 2H_2_O − CO]^+^, 432 [M + H − (Val + MeTyr + Phe + Ahp) − H_2_O]^+^, 420 [Ahp + Phe + MeTyr + H − H_2_O]^+^, 404 [M + H − (Val + MeTyr + Phe + Ahp) − H_2_O − CO]^+^, 386 [BA + Asp + Thr + Val + H]^+^, 308 [Phe(−N) + MeTyr + H]^+^, 297 [Asp + Thr + Val + H − H_2_O − CO]^+^, 243 [Ahp + Phe + H − H_2_O]^+^, 215 [Ahp + Phe + H − H_2_O − CO]^+^, 150 MeTyr immonium ion, 136 Tyr immonium ion, 120 Phe immonium ion.

**Figure 4:**
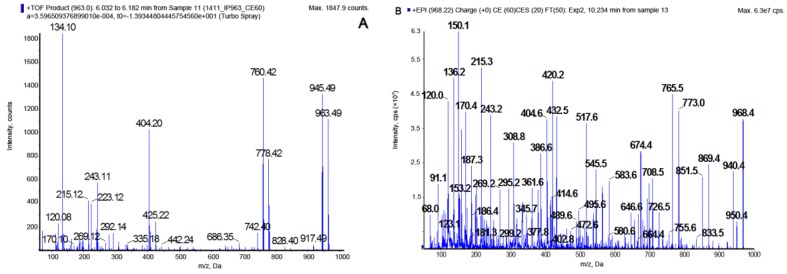
The product ion mass spectra of CP962 [Thr+Arg+Ahp+Phe+MePhe+Val]Asp+BA with precursor ion [M + H]+ at *m*/*z* 963. The spectra were recorded with application of a hybride quadrupole/time-of-flight mass spectrometer (QTOF) (**A**) and a hybride triple quadrupole/linear ion trap mass spectrometer (QTRAP) (**B**). The mass signals were assigned to the following fragments: 945 [M + H − H_2_O]^+^, 917 [M + H − H_2_O − CO]^+^, 846 [M + H − Val − H_2_O]^+^, 828 [M + H − Val − 2H_2_O]^+^, 778 [M + 2H − (BA + Asp)]^+^, 760 [M + 2H − (BA + Asp) − H_2_O]^+^, 742 [M + 2H − (BA + Asp) − 2H_2_O]^+^, 685 [M + H − (Val + MePhe) − H_2_O]^+^, 425 [BA + Asp + Thr + Arg + H − H_2_O]^+^, 404 [Ahp + Phe + MePhe + H − H_2_O]^+^, 297 [Asp + Thr + Val + H − H_2_O]^+^, 243 [Ahp + Phe + H − H_2_O]^+^, 215 [Ahp + Phe + H − H_2_O − CO]^+^, 134 MePhe immonium ion, 120 Phe immonium ion, 70-Arg.

Fractionation of *N. edaphicum* CCNP1411 crude extract (Figure S1) resulted in isolation of thirteen CPs. Structures were identified using a quadrupole/time of flight mass spectrometer and a triple quadrupole/linear ion trap mass spectrometer (Table 2). Structural elucidation of the peptides was based on fragmentation spectra with diagnostic ions, including immonium ions and a series of other fragment ions associated with specific residues. Depending on the residue in position 2, two types of spectra were obtained. Arg^2^-containing CPs (CPs-Arg^2^), gave pseudomolecular ions [M + H]^+^ at *m*/*z* 1049, 1021, 1019, 1007, 979, 993, 991, and 963. The Tyr^2^-containing peptides (CPs-Tyr^2^) were detected as dehydrated protonated molecules [M + H − H_2_O]^+^ at *m*/*z* 1010, 996, 982, 968, and 952, and the Tyr-immonium ion (*m*/*z* 136) was always present in their spectra. The putative planar structures of CPs detected in *N. edaphicum* CCNP1411 and their fragmentation spectra are presented in Figure 1, Figure 2, Figure 3 and Figure 4 and in supplementary information (Figures S2–S12). Amino acids at positions 1, 3, 4, 6, and 7 were found to be conserved and occupied by Thr^1^, Ahp^3^, Phe^4^, Val^6^, and Asp^7^, respectively. The ion peak corresponding to the longest sequence of residues common to all CP variants was observed in the spectra at *m*/*z* 297 [Asp + Thr + Val + H − H_2_O]^+^ or/and at *m*/*z* 269 [Asp + Thr + Val + H − H_2_O − CO]^+.^ The presence of butanoic acid (BA), hexanoic acid (HA), or octanoic acid (OA) in the side chain was mainly indicated by ion peaks formed by the cleavage of the corresponding fatty acid group (FA) and the exocyclic aspartic acid (Figure 3 and Figure 4; Figures S2–S12). As this cleavage produced a stable cyclic part of the molecule, the ions [M + H − (H_2_O) − (FA + Asp)]^+^ usually belonged to the most abundant ones. The residue in position 5 (i.e., *N*-MePhe, *N*-MeTyr or *N*-MeHty) was identified based on immonium ion peaks at *m*/*z* 134, 150 or 164, respectively, and peaks at *m*/*z* 404, 420, and 434 corresponding to [Ahp + Phe + (MePhe/MeTyr/MeHty) + H − H_2_O]^+.^ Ion peak at *m*/*z* 120, as well as peaks at 243 [Ahp + Phe + H − H_2_O]^+^ and 215 [Ahp + Phe + H − H_2_O − CO]^+^ confirmed the presence of Phe in position 4.

### 2.2. NMR Analysis

In order to confirm the structures, two CPs (i.e., CP962 and CP985) were purified in sufficient quantities for NMR spectroscopy. For both compounds the ^1^H-NMR spectra displayed the typical pattern of a peptide (i.e., doublet amide protons (δ_H_ 6.95–8.51 ppm) and a single amide methyl group (δ_H_ 2.71 ppm for CP985 and 2.84 ppm for CP962). The COSY, TOCSY, and HMBC experiments allowed assignment of NMR spin systems to Asp, Thr, Tyr, Ahp (3-amino-6-hydroxypiperid-2-one), Phe, MeTyr (*N*-methyl tyrosine), Val, and butanoic acid (BA) in the case of cyanopeptolin CP985 (Table 3, Figures S13–S18b). The presence of aromatic amino acid residues was recognized by the signals occurring in the aromatic region of the spectrum (δ_H_ 6.5–7.5 ppm). The AA’BB’ spin systems between two sets of tyrosine (Tyr-H2′/6′ and Tyr-H3′/5′, *J*_H,H_ = 8.4 Hz) and *N*-methyl tyrosine aromatic protons (MeTyr-H2′/6′ and MeTyr-H3′/5′, *J*_H,H_ = 8.4 Hz) indicated the presence of two *para*-di-substituted phenyl rings. The ^1^H-^13^C long range correlation from MeTyr-NH-CH_3_ group (δ_H_ 2.71 ppm) to the MeTyr-C2 atom (δ_C_ 61.3 ppm) revealed the presence of *N*-methyl tyrosine residue. Phenylalanine was found to be the third aromatic amino acid residue based on the COSY interaction between Phe-H2′/6′, Phe-H3′/5′, and Phe-H4′, and the HMBC correlation from two diastereotopic methylene protons Phe-3a (δ_H_ 2.88 ppm) and Phe-3b (δ_H_ 1.81 ppm) to the aromatic Phe-C2′/6′ carbons. The presence of Asp, Val, and Thr was confirmed by their characteristic spin systems in the COSY spectrum (Figure S17b). The macrocyclic ring closure between threonine and valine was verified by HMBC correlation between Thr-H3 (δ_H_ 5.36 ppm) and Val-C1 (δ_C_ 172.4 ppm) (Figure S15b). The presence of Ahp residue was detected by the characteristic signal of the OH proton (δ_H_ 5.99 ppm) and a broad singlet (δ_H_ 5.06 ppm) derived from H5 proton (Figure S13). The HMBC correlations from Ahp-C1 to Ahp-H5 and Ahp-H2 confirmed the cyclic nature of this residue. 

The COSY, TOCSY, and HMBC data allowed identification of amino acid residues in CP CP962 as Asp, Thr, Arg, Ahp, Phe, MePhe (*N*-methyl phenylalanine), Val, and BA, analogously to CP985 (Table 4, Figures S19–S24b). Two aromatic residues were found: phenylalanine and *N*-methyl phenylalanine. The occurrence of *N*-methyl group was established by the ^1^H-^13^C long range correlation from MePhe-NH-CH_3_ group (δ_H_ 2.84 ppm) to the MePhe-C2 atom. The typical ^13^C chemical shift of the guanidine quaternary carbon (δ_C_ 158.7 ppm) indicated the presence of Arg whose complete spin system was assigned based on TOCSY and COSY interactions (Figure S23b). The diagnostic regions of the TOCSY, ROESY and HMBC spectra of both cyanopeptolins analyzed are presented in corresponding figures in Supplementary.

### 2.3. Bioassays

Preparative chromatography resulted in separation of several fractions containing thirteen pure cyanopeptolin variants (Table 2). These peptides were evaluated for inhibition against four serine proteases and protein phosphatase 1. In addition, their effect on MCF-7 breast cancer cells was tested. However, only six CPs were isolated from *N. edaphicum* CCNP1411 in sufficient amounts to obtain quantitative results of the assays. None of the peptides were active against thrombin, elastase, and protein phosphatase 1, even at the highest concentration used in the study (45.4 μg/mL). However, all CPs with Arg in position 2 significantly reduced the activity of trypsin. The IC_50_ values of trypsin inhibitors were comparable and in the range of 0.24–0.26 μM. The CPs-Arg^2^ with *N-*MeTyr or *N-*MeHty in position 5 were also active against chymotrypsin, but the IC_50_ values were lower (IC_50_ = 3.1–3.8 μM). Chymotrypsin inhibition activity of CPs-Arg^2^ was not observed when position 5 was occupied by MePhe. All CPs-Tyr^2^ reduced activity of chymotrypsin and were inactive against other enzymes. For CP1027 and CP985, the IC_50_ was 0.26 μM. Following enzyme inhibition assays, all samples with Arg^2^-containing CP962 and Tyr^2^-containing CP985 (at 4.54 μg/mL) were analyzed by LC-MS/MS. The recovery was based on extracted mass chromatogram of parent ions. The enzymes did not cause any significant loss of the peptides; their contents were in the range from 94.6% to 97.7% of that in samples without the enzyme. 

**Figure 5:**
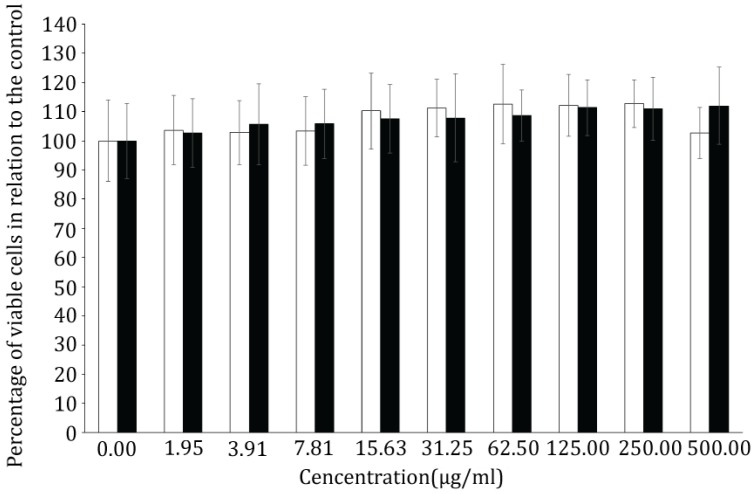
Viability of breast cancer cells MCF-7 treated for 24 h with Arg^2^-containing CP962 (white bars) and Tyr^2^-containing CP985 (black bars), isolated from *N. edaphicum* CCNP1411. Data are presented as percentage of the control, mean ± s.e.m, *n* = 3.

Due to limited amounts of pure peptides isolated from *N. edaphicum* CCNP1411, only Arg^2^-containing CP962 and Tyr^2^-containing CP985 were used in MTT (3-(4,5-dimethylthiazole-2-yl)-2,5-diphenyltetrazolium bromide) assay. After 24-h exposure, no cytotoxic effects on the MCF-7 cells were observed (Figure 5).

## 3. Discussion

Cyanobacteria from the *Nostoc* genus, especially the symbiotic strains and those living in waters from tropical regions, are important producers of bioactive compounds with potential biotechnological or pharmaceutical application [37,40,41,42,43]. One of the most prominent examples are cryptophycins, cyclic depsipeptides isolated from *Nostoc* sp. ATCC 53789 and GSV 224. They are promising candidates for anti-cancer drug development [44,45]. Another important *Nostoc* metabolite is cyanovirin-N, a small cyanobacterial lectin which blocks the entry of the enveloped viruses such as HIV, influenza, and Ebola [46].

In this study, the structures of CPs produced by *N. edaphicum* CCNP1411 from the Baltic Sea were elucidated based on the fragmentation spectra of their pseudomolecular ions (CPs-Arg^2^) (Figure 4; Figures S2–S8) or the dehydrated forms of the ions (CPs-Tyr^2^) (Figure 3; Figures S9–S12). The occurrence of the dehydrated pseudomolecular ion as a precursor ion in mass fragmentation spectra of Tyr^2^-(and Ile^2^)-containing aeruginopeptins has been previously reported [20,47]. It was suggested that the cleavage of an ester bond at Thr^1^ and subsequent dehydration resulted in the generation of linear peptides. For the two CPs, Arg^2^-containing CP962 and Tyr^2^-containing CP985, the composition and sequences of amino acids were confirmed by NMR spectroscopy. The results of the analyses were consistent with structure elucidation performed by tandem mass spectrometry. The core structures of these peptides were more similar to those identified in *Microcystis* [13,14,28,32], compared to those reported from other *Nostoc* isolates (Table 1). For example, instead of Arg^2^ or Tyr^2^ present in position 2 of the CPs identified in this work, nostopeptin and insulapeptolides from *N. minutum* and *N. insulare* possess Leu^2^ or Hph^2^ (homophenylalanine) [35,38]. Of the CPs identified in this study, only CP1006A (*m*/*z* 1007) was previously reported. This peptide, along with its chlorinated derivative (CP1040A) were found in *Microcystis* bloom and culture samples [14,48].

The structural diversity of CPs, specifically the residue in position 2, was found to have significant effect on activity of the peptides against serine proteases: trypsin and chymotrypsin [28,29,32]. The CP-type peptides with potent inhibitory activity against trypsin were characterized by the presence of basic amino acid (Arg or Lys), whereas in peptides active against chymotrypsin, position 2 was occupied by hydrophobic residues (Tyr, Phe, Hty or Leu). The same structure-activity relationship was observed in this study. Eight CPs-Arg^2^ inhibited the activity of trypsin with IC_50_ values of 0.24–0.26 µM, and five CPs-Tyr^2^ inhibited chymotrypsin with similar potency. In addition, the inhibitory activity of CPs produced by CCNP1411 seemed to be affected by the residue in position 5. Only those CPs-Arg^2^ which had *N*-MeTyr^5^ or *N*-MeHty^5^ in this position were active against chymotrypsin. However, to unequivocally prove the significance of this structure-activity relationship, more CPs should be tested. The fact that some CPs-Arg^2^, apart from strong inhibition of trypsin, are also active against chymotrypsin was previously documented by other authors [12,29,49]. The trypsin inhibition activity of CP-type peptides was suggested to be enhanced by the presence of isoleucine in position 6, instead of valine. Other modifications in the structure of Ahp-containing cyclic depsipeptides, such as the presence of chloride or sulfide groups, may also have an effect on enzyme inhibition activity [12]. 

Cyanobacteria produce many other Ahp-containing cyclic depsipeptides with inhibitory activity against proteases [28,29]. The majority of the peptides were active at micromolar concentrations, but some had even lower IC_50_ values. Symplocamide A, with citrulline in position 2, showed potent activity against chymotrypsin with IC_50_ of 0.38 µM, and was 200-times less active against trypsin (IC_50_ 80.2 µM) [29]. Symplocamide A also had cytotoxic activity to NCI H460 lung cancer cells and neuro-2A neuroblastoma cells [29]. Chymotrypsin was most potently inhibited by the glyceric acid 3′-*O*-phosphate-containing micropeptin T20 from *M. aeruginosa*, characterized by the presence of the Thr-Phe-Ahp sequence (IC_50_ 2.5 nM) [50]. Picomolar inhibition of trypsin was documented for CP 1020, which was also active against chymotrypsin, plasmin, human kallikrein, and factor XIa [32]. The structure of CP1020 differs from CP978 isolated in our study only in the presence of Glu in a side chain, instead of Asp. In enzymatic assays, CP978 and other CPs isolated from *N. edaphicum* CCNP1411 were less active than CP1020, but still belong to the most potent protease inhibitors among this class of compounds [12,28,29,32]. Ahp-containing depsipeptides were suggested to block the active center of trypsin or/and chymotrypsin, so the enzymes cannot cleave the peptide bonds at the carboxyl side of Arg or Tyr, respectively [25,28]. In this study, the Tyr^2^-containing CP985 and Arg^2^-containing CP962 exposed to proteases inhibited the activity of chymotrypsin or/and trypsin, but their concentrations remained almost unchanged. These results are in line with the hypothesis by Yamaki et al. [28] and confirm the blockage of the active centers of the enzymes by the peptides. 

Trypsin and chymotrypsin, the two enzymes inhibited by CPs from CCNP1411, are essential for food digestion. Their deregulation can also lead to a number of human diseases such as cancer, cardiovascular and inflammatory diseases. The molecules that modify activity of these proteases, and especially those that act selectively, are widely explored as agents of significant biotechnological and pharmaceutical potential [51]. As the new CP variants identified in *N. edaphicum* CCNP1411 inhibited the activity of trypsin or chymotrypsin at low concentrations and were inactive against the other tested enzymes and MCF-7 breast cancer cells, their possible use as therapeutic agents should be further explored.

Besides its therapeutic potential, this class of protease inhibitors was proven to be an important group of defense agents protecting cyanobacteria from grazers. Oscillapeptin J, CP SS and CP 1020 induced acute effects in small crustaceans [18,32,33]. However, CP SS was not toxic to the isolated rat hepatocytes [18], and oscillapeptin J did not induce any harmful effects in mice when administered intraperitoneally at concentrations up to 1000 µg/kg b.w. [52]. In the case of CP1020, induction of anti-inflammatory effects in human hepatoma cell line Huh7 was observed [53]. In addition, exposure of zebrafish eleuthero-embryos to CP1020 led to transcriptional alterations of genes involved in many important processes, including DNA damage recognition and repair, and circadian rhythm [54]. These findings, along with the acute toxicity of CP-type compounds observed in crustaceans suggest that their activity is not only related to the inhibition of digestive enzymes. 

## 4. Materials and Methods 

### 4.1. Culture Conditions

*Nostoc* cf. *edaphicum* CCNP 1411 (GenBank accession number KJ161445) was isolated in 2010 from the Gulf of Gdańsk, southern Baltic Sea, and established as monospecies culture by Dr. Justyna Kobos. Purification of the strain was carried out by multiple transfer to a liquid or/and solid (1% bacterial agar) Z8S medium [55]. To obtain a higher biomass of the cyanobacterium, the culture was grown in 5-L bottles at 22 °C ± 1 °C, at continuous light of 10–30 µE/m^2^/s. The collected biomass was lyophilized and kept at −20 °C until used.

### 4.2. Extraction and Isolation

Freeze-dried biomass of *N. edaphicum* (36 g) was extracted twice with 75% methanol (MeOH) (1 L) by vortexing for 30 min. Following centrifugation at 4,000 *g*, the extracts were pooled and diluted in water purified using ELGA PURELAB^®^ flex (Veoilia, London, UK) to adjust the concentration of MeOH to <10%. Then, the sample was loaded onto the preconditioned 120-g SNAP KP-C18-HS cartridge (Biotage Uppsala, Sweden). Sample components were eluted using an Isolera flash chromatography system (Biotage Uppsala, Sweden), with a step gradient (10–100% MeOH in water) with 3 column volumes of eluent at each step. Absorbance was monitored at 210 nm and 280 nm. The flow rate was 40 mL/min and 60-mL fractions were collected. Fraction composition was analyzed by UPLC-MS/MS. Fractions containing the same peptides were pooled, diluted with water to <10% MeOH, and concentrated on YMC C18 cartridges (20 mm ID × 2 cm; YMC, Gmbh, Dinslaken, Germany).

Six pooled fractions were further purified using preparative HPLC (Biotage Parallex Flex, Cardiff, UK) and Flex V3 software for instrument control and data acquisition. The separation was performed on XBridge Prep C18 column (5 µm CBD, 19 mm ID × 250 mm long; Waters, Elstree, UK) using a 30-min linear gradient from 15% to 80% acetonitrile in MilliQ water with 0.1% formic acid. Absorbance was monitored at 210 nm and 280 nm. The flow rate was 20 mL/min and 4-mL fractions were collected.

### 4.3. LC-MS/MS Analyses 

At each step of the extraction and isolation procedure, the content of the collected fractions was determined by UPLC-MS/MS. The system comprised a Waters Acquity Ultra performance LC coupled to a photodiode array detector (PDA) and a Xevo quadrupole time of flight mass detector (Waters, Elstree, UK). Samples were separated on an ethylene-bridged hybride BEH C18 column (2.1 mm ID × 100 mm; 1.7 µm, Waters) maintained at 40 °C. The mobile phase was Milli-Q water and acetonitrile (solvent B), both containing 0.1% formic acid. Separation was performed using gradient elution (0.3 mL/min) from 20% to 70% B over 10 min, followed by a 100% B wash step and re-equilibration. Data was acquired in positive ion electrospray scanning from *m*/*z* 50 to 2000 with a scan time of 2 s and inter-scan delay of 0.1 s. The capillary and cone voltages were set at 0.7 kV and 25 V, respectively. The desolvation gas was maintained at 400 L/h at a temperature of 300 °C. The cone gas was set at 50 L/h with a source temperature of 80 °C. Instrument control, data acquisition and processing were achieved using MassLynx v4.1 (Waters, Milford, MA, USA). 

Structures of cyanopeptolins were additionally characterized using Agilent 1200 HPLC (Agilent Technologies, Waldbronn, Germany) coupled to a hybrid triple quadrupole/linear ion trap mass spectrometer QTRAP5500 (Applied Biosystems MDS Sciex, Concord, ON, Canada). Peptides were separated on Zorbax Eclipse XDB-C18 column (4.6 mm ID × 150 mm, 5 μm; Agilent Technologies, Santa Clara, CA, USA) column. A gradient elution (0.6 mL/min) was applied with mobile phase composed of 5% acetonitrile in MilliQ water and acetonitrile (solvent B), both containing 0.1% formic acid. The gradient started at 15% B and went to 50% B within 5 min. The content of phase B was then increased to 100% within the next 3 min and kept at that level for 10 min before returning to the starting conditions. The QTRAP MS/MS system was operated in the positive mode, with turbo ion source voltage set at 5.5 kV and temperature at 550 °C. For ions within the *m*/*z* range 500–1250 and signal intensity above the threshold of 500,000 cps, fragmentation spectra were acquired within a range 50–1000 Da, at collision energy of 60 V and declustering potential set at 80 eV. Data acquisition and processing were accomplished with the Analyst^®^ Sofware (version 1.5.1, Applied Biosystems, Concord, ON, Canada).

### 4.4. NMR Analyses

1D ^1^H-NMR and 2D homo- and heteronuclear 2D NMR (COSY, TOCSY, ROESY, HSQC, and HMBC) were acquired on a Bruker Avance III spectrometers, 500 MHz and 700 MHz. Spectra were recorded in DMSO-d_6_. NMR data were processed and analyzed by TopSpin (Bruker, Billerica, MA, USA) and SPARKY software (3.114, Goddard and Kneller, freeware https://www.cgl.ucsf.edu/home/sparky). 

### 4.5. Enzyme Inhibition Assay

The chymotrypsin and trypsin inhibition assays were performed following the procedures of Ploutno and Carmeli [41]. The α-chymotrypsin from bovine pancreas (C4129), trypsin from porcine pancreas (T0303), aprotinin (1.5–200 μg/mL) as enzyme inhibitor, *N*-Suc-Gly-Gly-*p*-nitroanilide and *N*-α-benzoyl-dl-arginine-*p*-nitroanilide hydrochloride (BAPNA) as chymotrypsin and trypsin substrates, respectively were used. Enzymes (0.1 mg/mL) and substrate (2 mM) were dissolved in the same buffer (50 mM Tris-HCl, 100 mM NaCl, 1 mM CaCl_2_, pH 7.5). The thrombin inhibition assay was performed according to Ocampo and Bennet [56]. Thrombin (T4648) was dissolved in buffer (0.5 mg/mL; 0.2 M Tris-HCl; pH 8.0); substrate (*N*-*p*-tosyl-Gly-l-Pro-l-Lys-*p*-nitroanilide acetate salt, 0.5 mg/mL) and inhibitor (4-(2-aminoethyl)benzenesulfonyl fluoride hydrochloride (AEBSF; 60–2400 μg/mL)) were dissolved in MilliQ water. The elastase inhibition assay was performed according to Kwan et al. [57]. The enzyme (75 μg/mL; E0258) from porcine pancreas, substrate (2 mM; *N*-Suc-Ala-Ala-Ala-*p*-nitroanilide) and inhibitor (elastatinal; 5–125 μg/mL) were dissolved in buffer (0.2 M Tris-HCl; pH 8.0). All enzymes, substrates and inhibitors used for proteases inhibition assays were from Sigma-Aldrich (St. Louis, MO, USA). Protein phosphatase 1 inhibition assay was performed according to the procedure described by Rapala et al. [58]. PP1 was from England Biolabs, Hitchin, UK (754S) and the substrate, *p*-nitrophenyl phosphate disodium salt hexahydrate (*p*-NPP), was from Sigma-Aldrich (Irvine, UK). Microcystin MC-LR (0.125–4.0 ng/mL) from Enzo Life Sciences, Lausen, Switzerland was used as inhibitor. The enzyme was dissolved in buffer solution A (50 mM Tris at pH 7.4, 1 mg/mL bovine serum albumin (BSA; Sigma -Aldrich, St. Louis, MO, USA), 1 mM MnCl_2_, 2 mM dithiotreitol (DTT; Sigma-Aldrich, St. Louis, MO, USA)). *p*-NPP (5.5 mg/mL) was dissolved in buffer solution B (50 mM Tris, pH 8.1, 0.5 mg/mL BSA, 20 mM MgCl_2_ × 6H_2_O, 200 mM MnCl_2_ × 4H_2_O). All enzyme inhibition assays were performed in a 96 multi-well plate at 37 °C, in triplicate. The absorbance of the reaction mixtures was measured at 405 nm using a microplate reader (Molecular Devices, Sunnyvale, CA, USA). After trypsin and chymotrypsin inhibition assays, the samples with and without the enzymes and containing the highest concentration of CP962 and CP985 (45.4 µg/mL) were analyzed by LC-MS/MS. The content of the peptides was determined based of the peak area of the extracted ions.

### 4.6. Cytotoxicity Assay

For the test, two CPs were selected: Arg^2^-containing CP962 and Tyr^2^-containing CP985. MCF-7 breast cancer cells were seeded at 7.5 × 10^3^ cells/100 μL in a 96-well plates and incubated at 37 °C, 5% CO_2_ for 24 h. The cells were then treated for a further 24 h with CP962 and CP985 (0 to 500 μg/mL). After 24 h, sterile-filtered 3-(4,5-dimethylthiazol-2-yl)-2,5-diphenyltetrazolium bromide solution (MTT; 1 mg/mL) was added to each well. After 4 h incubation at 37 °C in the dark, the MTT solution was removed and formazan crystals solubilized in DMSO. The plates were shaken for 20 min, in the dark, at room temperature and absorbance was measured at 560 nm (Synergy/HT, BIOTEK, Wnooski, VT, USA). For each CP, three independent experiments were carried out and each treatment consisted of six replicates per plate. Bar charts were used to represent the viability of MCF-7 cells treated with CP962 and CP985, compared to the control (i.e., untreated cells) that represented 100% cell viability. 

## 5. Conclusions

*Nostoc edaphicum* CCNP1411 isolated from the Gulf of Gdańsk (southern Baltic) produces at least thirteen CPs, including twelve variants reported here for the first time. The structures of the peptides are different from other Ahp-containing cyclic depsipeptides previously found in *Nostoc*. The activity of the peptides was mainly determined by the presence of Arg^2^ or Tyr^2^ in Ahp-adjacent position. The fact that trypsin and chymotrypsin did not degrade the tested CPs constitutes an additional evidence for enzyme inactivation by the peptides. Neither of the CP inhibited thrombin, elastase, and protein phosphatase 1; CP962 and CP985 also showed no cytotoxic effects on MCF-7 breast cancer cells. The CPs produced by *N. edaphicum* CCNP1411, as peptidic structures with selective and potent proteases inhibiting activity, are potential lead compounds in drug discovery process.

## Figures and Tables

**Table 1:** Cyanopeptolin-type peptides identified in cyanobacteria from *Nostoc* genus.

Molecular Mass	Peptide Name	Structure	Enzyme Inhibition	References
921	Nostopeptin BN920	[Thr+Leu+Ahp ^1^ +Phe+MeTyr+Val]Gln+Ac ^2^	Chymotrypsin (IC_50_ 0.11 μM)	[36]
926	Nostopeptin B	[Hmp ^3^ +Leu+Ahp+Ile+MeTyr+Ile]Gln+Ac	Elastase (IC_50_ 11.0 μg/mL) Chymotrypsin (IC_50_ 1.6 μg/mL)	[35]
937	Nostopeptin A	[Hmp+Leu+Ahp+Ile+MeTyr+Ile]Gln+BA ^4^	Elastase (IC_50_ 1.3 μg/mL) Chymotrypsin (IC_50_ 1.4 μg/mL)	[35]
942	Insulapeptolide A	[Hmp+Leu+Ahp+Ile+MeTyr+Val]Cit ^5^ +Ac	HLE ^10^ (IC_50_ 0.14 μM) *	[38]
956	Insulapeptolide B	[Hmp+Leu+Ahp+Leu+MeTyr+Ile]Cit+Ac	HLE (IC_50_ 0.10 μM) *	
956	Insulapeptolide C	[Hmp+Leu+Ahp+Ile+diMeTyr ^6^ +Val]Cit+Ac	HLE (IC_50_ 0.090 μM) *	
970	Insulapeptolide D	[Hmp+Leu+Ahp+Ile+diMeTyr+Ile]Cit+Ac	HLE (IC_50_ 0.085 μM) *	
991	Insulapeptolide G	[Thr+Hph ^7^ +Ahp+Thr+MePhe+Val]Ser+Pro+BA	HLE (IC_50_ 3.5 μM) *	
1005	Insulapeptolide H	[Thr+Hph+Ahp+Thr+MeTyr+Ile]Ser+Pro+BA	HLE (IC_50_ 2.7μM) *	
1007	Insulapeptolide F	[Thr+Hph+Ahp+Thr+MeTyr+Val]Ser+Pro+BA	HLE (IC_50_ 1.6 μM) *	
1021	Insulapeptolide E	[Thr+Hph+Ahp+Thr+MeTyr+Ile]Ser+Pro+BA	HLE (IC_50_ 3.2 μM) *	
1116	Nostocyclin	[Thr+Hse ^8^ +Ahp+Phe+MeTyr+Val]Hse+Ile+Hpla ^9^	PP1 (IC_50_ 64.0 μM)	[34]

* activity of extracts; ^1^ Ahp 3-amino-6-hydroxy-2-piperidone; ^2^ Ac acetic acid; ^3^ Hmp 3-hydroxy-4-methyl-proline; ^4^ BA butanoic acid; ^5^ Cit citrulline; ^6^ diMeTyr *N*,*O*-dimethyltyrosine; ^7^ Hph homophenylalanine; ^8^ Hse homoserine; ^9^ Hpla p-hydroxyphenyllactic acid; ^10^ HLE human leukocyte elastase.

**Table 2:** Cyanopeptolins identified in *Nostoc edaphicum* CCNP 1411. The activities of the peptides were assessed in serine proteases (trypsin, chymotrypsin, elastase, and thrombin) and protein phosphatases 1 (PP 1) inhibition assays (- not active; */** small/medium activity; *m*/*z* of precursor ions: [M + H]^+^ for CPs-Arg^2^ and [M + H − H_2_O]^+^ for CPs-Tyr^2^.

Cyanopeptolin CP	*m*/*z*	Structure	Enzyme Inhibition (IC_50_ [µM])				
			Chymotrypsin	Trypsin	Elastase	Thrombin	Protein Phosphatase 1
CP 1048	1049	[Thr+Arg+Ahp+Phe+MeHty+Val]Asp+OA	*	**	-	-	-
CP 1020	1021	[Thr+Arg+Ahp+Phe+MeHty+Val Asp+HA	3.1	0.25	-	-	-
CP 1018	1019	[Thr+Arg+Ahp+Phe+MePhe+Val]Asp+ OA	-	0.24	-	-	-
CP 1006	1007	[Thr+Arg+Ahp+Phe+MeTyr+Val]Asp+HA	*	**	-	-	-
CP 992	993	[Thr+Arg+Ahp+Phe+MeHty+Val]Asp+BA	3.5	0.24	-	-	-
CP 990	991	[Thr+Arg+Ahp+Phe+MePhe+Val]Asp+HA	-	**	-	-	-
CP 978	979	[Thr+Arg+Ahp+Phe+MeTyr+Val]Asp+BA	3.8	0.26	-	-	-
CP 962	963	[Thr+Arg+Ahp+Phe+MePhe+Val]Asp+BA	-	**	-	-	-
CP 1027	1010	[Thr+Tyr+Ahp+Phe+MeHty+Val]Asp+HA	0.26	-	-	-	-
CP 1013	996	[Thr+Tyr+Ahp+Phe+MeTyr+Val]Asp+HA	**	-	-	-	-
CP 999	982	[Thr+Tyr+Ahp+Phe+MeHty+Val]Asp+BA	**	-	-.	-	-
CP 985	968	[Thr+Tyr+Ahp+Phe+MeTyr+Val]Asp+BA	0.26	-	-	-	-
CP 969	952	[Thr+Tyr+Ahp+Phe+MePhe+Val]Asp+BA	**	-	-	-	-

**Table 3:** NMR Spectroscopic data (700 MHz, DMSO-*d*_6_) for cyanopeptolin CP985 [Thr+Tyr+Ahp+Phe+MeTyr+Val]Asp+BA.

Unit	Position	δ_C_	δ_H_ (*J* in Hz)	ROESY	HMBC *^a^*
**BA**	1 2 3 4	172.9, C 37.7, CH_2_ 19.2, CH_2_ 14.2, CH_3_	2.11, m 1.56, m 0.89, t (7.2, 7.2)	Asp-NH	BA-1, BA-3, BA-4 BA-1, BA-2, BA-4 BA-2
**Asp**	1 2 3a 3b 4 NH	171.7, C 49.9, CH 36.1, CH_2_ 172.4, C	4.58, dd (8.0, 5.0) 2.65, m 2.46, m 8.26, m	Thr-NH	Asp-4
**Thr^1^**	1 2 3 4 NH	168.9, C 54.7, CH 72.6, CH 18.0, CH_3_	4.52, d (10.0) 5.36, q (6.8, 6.9, 6.8) 1.16, d (7.2) 7.52, d (9.3)	Tyr-NH Tyr-NH Asp-2	Thr-1, Asp-1 Val-1 Thr-2, Thr-3
**Tyr^2^**	1 2 3a 3b 1’ 2′/6′ 3′/5′ 4′ NH	169.9, C 54.2, CH 35.5, CH_2_ 128.5, C 130.1, CH 115.5, CH 156.1, C	4.31, m 3.11, m 2.55, m 6.89, d (8.4) 6.58, d (8.4) 8.46, d (8.8)	Ahp-NH Thr-2, Thr-3	Tyr-2, Tyr-1′ Tyr-1′ Tyr-4′ Thr-1
**Ahp^3^**	1 2 3 4 5 NH OH	169.0, C 49.4, CH 22.0, CH_2_ 21.9, CH_2_ 74.1, CH	3.62, m 2.41, m 1.64, m 5.06, brs 7.06, d (8.8) 5.99, d (3.1)	Ahp-NH Phe-3a, Phe-3b Tyr-2, Ahp-3	Ahp-1, Ahp-3 Ahp-2 Ahp-1 Tyr-1
**Phe^4^**	1 2 3a 3b 1′ 2′/6′ 3′/5′ 4′	170.8, C 50.7, CH 35.8, CH_2_ 137.2, C 129.9, CH 128.2, CH 126.7, CH	4.76, dd (7.1, 4.6) 2.88, t (12.9, 12.9) 1.81, dd (10.4, 3.9) 6.84, d (7.0) 7.19, t (7.3, 7.3) 7.14, d (7.0)	Ahp-5	Phe-2, Phe-1′, Phe-2′/6′ Phe-3a, Phe-3b, Phe-4′ Phe-1′ Phe C2′/6′
**MeTyr^5^**	1 2 3 1′ 2′/6′ 3′/5′ 4′ NCH_3_ OH	169.4, C 61.3, CH 33.3, CH_2_ 128.2, C 130.9, CH 115.5, CH 156.7, C 33.3, CH_3_	4.89, dd (8.9, 2.5) 3.10, m 7.00, d (8.4) 6.78, d (8.4) 2.71, s 9.33, s	Val-NH	MeTyr-1′ MeTyr-1′, MeTyr-3′/5′ MeTyr-2′/6′, MeTyr-4′ MeTyr-2, Phe-1 MeTyr-3′/5′, MeTyr-4′
**Val^6^**	1 2 3 4 5 NH	172.4, C 56.3, CH 31.4, CH 19.7, CH_3_ 17.7, CH_3_	4.63, dd (4.9, 4.6) 2.02, m 0.84, d (6.6) 0.71, d (6.6) 7.39, d (9.7)	MeTyr-2	Val-2, Val-3, Val-5 Val-2, Val-3, Val-4 MeTyr-1

*^a^* HMBC correlations are given from proton(s) stated to the indicated carbon atom.

**Table 4:** NMR Spectroscopic data (700 MHz, DMSO-d_6_) for cyanopeptolin CPL962 [Thr+Arg+Ahp+Phe+MePhe+Val]Asp+BA.

Unit	Position	δ_C_	δ_H_ (*J* in Hz)	ROESY	HMBC *^a^*
**BA**	1 2 3 4	172.2, C 37.4, CH_2_ 19.3, CH_2_ 14.1, CH_3_	2.08, m 1.52, m 0.87, t (7.4, 7.4)	Asp-NH	BA-1, BA-3, BA-4 BA-1, BA-2, BA-4 BA-2
**Asp**	1 2 3a 3b 4 NH	173.7, C 50.8, CH 40.1, CH_2_ 175.3, C	4.54, m 2.51, m 2.12, m 8.00, d (8.0)	Thr-NH	Asp-2, Asp-4 BA-1
**Thr^1^**	1 2 3 4 NH	169.9, C 55.0, CH 72.3, CH 17.9, CH_3_	4.58, d (9.0) 5.30, q (6.8, 6.5, 6.8) 1.15, d (6.5) 7.23, d (7.6)	Arg-NH Arg-NH Asp-2	Thr-1 Thr-4, Val-1 Thr-2, Thr-3
**Arg^2^**	1 2 3 4 5 6 NH	170.4, C 49.0, CH 26.4, CH_2_ 24.5, CH_2_ 39.8, CH_2_ 158.7, C	4.15, m 1.88, m 1.46, m 2.95, m 8.5, d (8.7)	Ahp-NH Thr-2, Thr-3	Thr-1
**Ahp^3^**	1 2 3 4 5 NH OH	169.6, C 48.6, CH 21.7, CH_2_ 22.0, CH_2_ 74.2, CH	3.65, m 2.43, m 1.68, m 5.03, brs 6.95, d (9.7) 6.01, d (2.6)	Phe-3a, Phe-3b Arg-2, Ahp-3	Ahp-1, Ahp-3 Ahp-1 Arg-1
**Phe^4^**	1 2 3a 3b 1′ 2′/6′ 3′/5′ 4′	170.0, C 50.6, CH 35.6, CH_2_ 137.1, C 129.8, CH 128.2, CH 126.7, CH	4.74, dd (7.2, 3.5) 2.86, m 1.69, m 6.78, d (7.1) 7.17, t (7.1, 7.1) 7.13, d (7.8)	Ahp-5	Phe-1, Phe-3a, Phe-3b Phe-1′ Phe-3a, Phe-3b, Phe-4′ Phe-1′ Phe-3′/5′
**MePhe^5^**	1 2 3 1′ 2′/6′ 3′/5′ 4′ NCH_3_	169.4, C 60.9, CH 34.4, CH_2_ 138.4, C 130.0, CH 129.1, CH 127.2, CH 35.6, CH_3_	5.02, m 3.23, m 7.24, d (7.6) 7.41, t (7.7, 7.7) 7.32, d (7.5) 2.84, s	Val-NH	MePhe-1′ MePhe-1′ MePhe-4′ MePhe-3′/5′ MePhe-2
**Val^6^**	1 2 3 4 5 NH	172.6, C 56.4, CH 31.6, CH 19.7, CH_3_ 17.8, CH_3_	4.71, dd (5.2, 4.2) 2.05, m 0.88, d (6.9) 0.76, d (6.9) 7.41, d (7.7)	MePhe-2	Val-3 Val-2, Val-3, Val-5 Val-2, Val-3, Val-4 MePhe-1

*^a^* HMBC correlations are given from proton(s) stated to the indicated carbon atom.

## Supplementary Materials

The following are available online at http://www.mdpi.com/1660-3397/16/7/220/s1. Figure S1: LC-MS/MS chromatogram of cyjanopeptolins (CPs) in crude extract from *Nostoc edaphicum* CCNP1411 (**A**) and chromatograms of isolated peptides: CP962 (**B**) and CP985 (**C**); Figure S2: Chemical structure (**A**) and product ion mass spectra of cyanopeptolin CP1049 [Thr+Arg+Ahp+Phe+MeHty+Val]Asp+OA with precursor ion [M + H]^+^ at *m*/*z* 1049. The spectra were recorded with application of a hybride quadrupole/time-of-flight mass spectrometer (QTOF) (**B**) and a hybride triple quadrupole/linear ion trap mass spectrometer (QTRAP) (**C**). The mass signals were assigned to the following fragments: 1031 [M + H − H_2_O]^+^, 1003 [M + H − H_2_O − CO]^+^, 932 [M + H − Val − H_2_O]^+^, 914 [M + H − Val − 2H_2_O]^+^, 808 [M + 2H − (Asp + OA)]^+^, 790 [M + 2H − (Asp + OA) − H_2_O]^+^, 772 [M + 2H − (Asp + OA) − 2H_2_O]^+^, 741 [M + H − (Val + MeHty) − H_2_O]^+^, 673 [M + 2H − Val − (Asp + OA) − 2H_2_O]^+^, 481 [OA+Asp + Thr + Arg + H − H_2_O]^+^, 434 [Ahp + Phe + MeHty + H − H_2_O]^+^, 338 [Arg + Thr + Val + H − H_2_O]^+^, 322 [Phe(−N) + MeHty + H]^+^, 297 [Asp + Thr + Val + H − H_2_O]^+^, 243 [Ahp + Phe + H − H_2_O]^+^, 215 [Ahp + Phe + H − H_2_O − CO]^+^, 164 MeHty immonium ion, 120 Phe immonium ion, 70-Arg; Figure S3: Chemical structure (**A**) and product ion mass spectra of cyanopeptolin CP1020 [Thr+Arg+Ahp+Phe+MeHty+Val]Asp+HA with precursor ion [M + H]^+^ at *m*/*z* 1021. The spectra were recorded with application of QTOF (**B**) and QTRAP (**C**) mass spectrometers. The mass signals were assigned to the following fragments: 1003 [M + H − H_2_O]^+^, 975 [M + H − H_2_O − CO]^+^, 886 [M + H − Val − 2H_2_O]^+^, 808 [M + 2H − (Asp + HA)]^+^, 790 [M + 2H − (Asp + HA) − H_2_O]^+^, 772 [M + 2H − (Asp + OA) − 2H_2_O]^+^, 713 [M + H − (Val + MeHty) − H_2_O]^+^, 691 [M + 2H − Val − (Asp + HA) − H_2_O]^+^, 673 [M + 2H − Val − (Asp + HA) − 2H_2_O]^+^, 453 [HA + Asp + Thr + Arg + H − H_2_O]^+^, 434 [Ahp + Phe + MeHty + − H_2_O]^+^, 338 [Arg + Thr + Val + H − H_2_O]^+^, 322 [Phe(-N) + MeHty + H]^+^, 297 [Asp + Thr + Val + H − H_2_O]^+^, 243 [Ahp + Phe + H − H_2_O]^+^, 215 [Ahp + Phe + H − H_2_O − CO]^+^, 164 MeHty immonium ion, 120 Phe immonium ion, 70-Arg; Figure S4: Chemical structure (**A**) and product ion mass spectra of cyanopeptolin CP1018 [Thr+Arg+Ahp+Phe+MePhe+Val]Asp+OA with precursor ion [M+H]^+^ at *m*/*z* 1019. The spectra were recorded with application of QTOF (**B**) and QTRAP (**C**) mass spectrometers. The mass signals were assigned to the following fragments: 1001 [M + H − H_2_O]^+^, 983 [M + H − 2H_2_O]^+^, 973 [M + H − H_2_O − CO]^+^, 902 [M + H − Val − H_2_O]^+^, 884 [M + H − Val − 2H_2_O]^+^, 778 [M + 2H − (Asp + OA)]^+^, 760 [M + 2H − (Asp + OA) − H_2_O]^+^, 742 [M + 2H − (Asp + OA) − 2H_2_O]^+^, 661 [M + 2H − Val − (Asp+OA) − H_2_O]^+^, 643 [M + 2H − Val − (Asp + OA) − 2H_2_O]^+^, 481 [OA + Asp + Thr + Arg + H − H_2_O]^+^, 404 [Ahp + Phe + MePhe + H − H_2_O]^+^, 338 [Arg + Thr + Val + H − H_2_O]^+^, 308 [Phe(-N) + MeTyr + H]^+^, 297 [Asp + Thr + Val + H − H_2_O]^+^, 243 [Ahp + Phe + H − H_2_O]^+^, 215 [Ahp + Phe + H − H_2_O − CO]^+^, 134 MePhe immonium ion, 120 Phe immonium ion, 70-Arg; Figure S5: Chemical structure (**A**) and product ion mass spectra of cyanopeptolin CP1006 [Thr+Arg+Ahp+Phe+MeTyr+Val]Asp+HA with precursor ion [M + H]^+^ at *m*/*z* 1007. The spectra were recorded with application of QTOF (**B**) and QTRAP (**A**) mass spectrometers. The mass signals were assigned to the following fragments: 989 [M + H − H_2_O]^+^, 961 [M + H − H_2_O − CO]^+^, 872 [M + H − Val − 2H_2_O]^+^, 794 [M + 2H − (Asp + HA)]^+^, 776 [M + 2H − (Asp + HA) − H_2_O]^+^, 766 [ M + 2H − (Asp + HA) − CO]^+^, 758 [M + 2H − (Asp + HA) − 2H_2_O]^+^, 713 [M + H − (Val + MeHTyr) − H_2_O]^+^, 659 [M + 2H − Val − (Asp + HA) − 2H_2_O]^+^, 453 [HA + Asp + Thr + Arg + H − H_2_O]^+^, 420 [Ahp + Phe + MeTyr + H − H_2_O]^+^, 338 [Arg + Thr + Val + H − H_2_O]^+^, 308 [Phe(−N) + MeTyr + H]^+^, 297 [Asp + Thr + Val + H − H_2_O]^+^, 243 [Ahp + Phe + H − H_2_O]^+^, 215 [Ahp + Phe + H − H_2_O − CO]^+^, 150 MeTyr immonium ion, 120 Phe immonium ion, 70-Arg; Figure S6: Chemical structure (**A**) and product ion mass spectra of cyanopeptolin CP992 [Thr+Arg+Ahp+Phe+MeHty+Val]Asp+BA with precursor ion [M + H]^+^ at *m*/*z* 993. The spectra were recorded with application of QTOF (**B**) and QTRAP (**C**) mass spectrometers. The mass signals were assigned to the following fragments: 975 [M + H − H_2_O]^+^, 947 [M + H − H_2_O − CO]^+^, 858 [M + H − Val −-2H_2_O]^+^, 808 [M + 2H − (Asp + BA)]^+^, 790 [M + 2H − (Asp + BA) − H_2_O]^+^, 772 [M + 2H − (Asp + BA) − 2H_2_O]^+^, 673 [M + 2H − Val − (Asp + BA) − 2H_2_O]^+^, 434 [Ahp + Phe + MeHty + H − H_2_O]^+^, 425 [BA + Asp + Thr + Arg + H − H_2_O]^+^, 338 [Arg + Thr + Val + H − H_2_O]^+^, 322 [Phe(−N) + MeHty + H]^+^, 243 [Ahp + Ph + H − H_2_O]^+^, 215 [Ahp + Phe + H − H_2_O − CO]^+^, 164 MeHty immonium ion, 120 Phe immonium ion, 70-Arg; Figure S7: Chemical structure (**A**) and product ion mass spectra of cyanopeptolin CP990 [Thr+Arg+Ahp+Phe +MePhe+Val]Asp+HA with precursor ion [M + H]^+^ at *m*/*z* 991. The spectra were recorded with application of QTOF (**B**) and QTRAP (**C**) mass spectrometers. The mass signals were assigned to the following fragments: 973 [M + H − H_2_O]^+^, 945 [M + H − H_2_O − CO]^+^, 856 [M + H − Val − 2H_2_O]^+^, 778 [M + 2H − (Asp + HA)]^+^, 760 [M + 2H − (Asp + HA) − H_2_O]^+^, 750 [M + 2H − (Asp + HA) − CO]^+^, 742 [M + 2H-(Asp + HA) − 2H_2_O]^+^, 643 [M + 2H − Val − (Asp + HA) − 2H_2_O]^+^, 453 [HA + Asp + Thr + Arg + H − H_2_O]^+^, 404 [Ahp + Phe + MePhe + H − H_2_O]^+^, 338 [Arg + Thr + Val + H − H_2_O]^+^, 297 [Asp + Thr + Val + H − H_2_O]^+^, 243 [Ahp+Phe+H-H_2_O]^+^, 215 [Ahp+Phe+H-H_2_O-CO]^+^, 134 MePhe immonium ion, 120 Phe immonium ion, 70-Arg; Figure S8: Chemical structure (**A**) and product ion mass spectra of cyanopeptolin CP978 [Thr+Arg+Ahp+Phe+MeTyr+Val]Asp+BA with precursor ion [M+H]^+^ at *m*/*z* 979. The spectra were recorded with application of QTOF (**B**) and QTRAP (**C**) mass spectrometers. The mass signals were assigned to the following fragments: 961 [M + H − H_2_O]^+^, 933 [M + H − H_2_O − CO]^+^, 844 [M + H − Val − 2H_2_O]^+^, 794 [M + 2H − (Asp + BA)]^+^, 776 [M + 2H − (Asp + BA) − H_2_O]^+^, 758 [M + 2H − (Asp + BA) − 2H_2_O]^+^, 659 [M + 2H − Val − (Asp + BA) − 2H_2_O]^+^, 425 [BA + Asp + Thr + Arg + H − H_2_O]^+^, 420 [Ahp + Phe + MeTyr + H − H_2_O]^+^, 338 [Arg + Thr + Val + H − H_2_O]^+^, 308 [Phe(−N) + MeTyr + H]^+^, 243 [Ahp + Phe + H − H_2_O]^+^, 215 [Ahp + Phe + H − H_2_O − CO]^+^, 150 MeTyr immonium ion, 120 Phe immonium ion, 70-Arg; Figure S9: Chemical structure (**A**) and product ion mass spectra of cyanopeptolin CP1027 [Thr+Tyr+Ahp+Phe+MeHty+Val]Asp+HA with precursor ion [M+H-H_2_O]^+^ at *m*/*z* 1010. The spectra were recorded with application of QTOF (**B**) and QTRAP (**C**) mass spectrometers. The mass signals were assigned to the following fragments: 992 [M + H − 2H_2_O]^+^, 982 [M + H − H_2_O − CO]^+^, 964 [M + H − 2H_2_O − CO]^+^, 911 [M + H − Val − H_2_O]^+^, 893 [M + H − Val − 2H_2_O]^+^, 819 [M + H − MeHty − H_2_O]^+^, 797 [M + 2H – (Asp + HA) − H_2_O]^+^, 779 [M + 2H − (Asp + HA) − 2H_2_O]^+^, 751 [M + 2H − (Asp + HA) − 2H_2_O − CO]^+^, 702[M + H − (Val + MeHty) − H_2_O]^+^, 674 [M + H − (Val + MeHty) − H_2_O − CO]^+^, 460 [M + H − (Val + MeHty + Phe + Ahp) − H_2_O]^+^, 442 [M + H − (Val + MeHty + Phe + Ahp) − 2H_2_O]^+^, 434 [Ahp + Phe + MeHty +H − H_2_O]^+^, 322 [Phe(−N) + MeHty + H]^+^, 297 [Asp + Thr+ Val +H − H_2_O]^+^, 243 [Ahp +Phe + H − H_2_O]^+^, 215 [Ahp + Phe + H − H_2_O − CO]^+^, 164 MeHty immonium ion, 136 Tyr immonium ion, 120 Phe immonium ion; Figure S10: Chemical structure (**A**) and product ion mass spectra of cyanopeptolin CP1013 [Thr+Tyr+Ahp+Phe+MeTyr+Val]Asp+HA with precursor ion [M+H-H_2_O]^+^ at *m*/*z* 996. The spectra were recorded with application of QTOF (**B**) and QTRAP (**C**) mass spectrometers. The mass signals were assigned to the following fragments: 978 [M + H − 2H_2_O]^+^, 968 [M + H − H_2_O − CO]^+^, 897 [M + H − Val − H_2_O]^+^, 879 [M + H − Val − 2H_2_O]^+^, 819 [M + H − MeTyr − H_2_O]^+^, 783 [M + 2H − (Asp + HA) − H_2_O]^+^, 765 [M + 2H − (Asp + HA) − 2H_2_O]^+^, 736 [M + H − (Asp + HA) − 2H_2_O − CO]^+^, 720 [M + H − (Val + MeTyr) − H_2_O]^+^, 702 [M + H − (Val + MeTyr) − 2H_2_O]^+^, 666 [M + 2H − Val − (Asp + HA) − 2H_2_O]^+^, 460 [M + H − (Val + MeTyr + Phe + Ahp) − H_2_O]^+^, 420 [Ahp + Phe + MeTyr + H − H_2_O]^+^, 432 [M + H − (Val + MeTyr + Phe + Ahp) − H_2_O − CO]^+^, 414 [HA + Asp + Thr + Val + H]^+^, 297 [Asp + Thr + Val + H − H_2_O]^+^, 243 [Ahp + Phe + H − H_2_O]^+^, 215 [Ahp + Phe+ H − H_2_O − CO]^+^, 150 MeTyr immonium ion, 136 Tyr immonium ion, 120 Phe immonium ion; Figure S11. Chemical structure (**A**) and product ion mass spectra of cyanopeptolin CP999 [Thr+Tyr+Ahp+Phe+MeHty+Val]Asp+BA with precursor ion [M+H-H_2_O]^+^ at *m*/*z* 982. The spectra were recorded with application of QTOF (**B**) and QTRAP (**C**) mass spectrometers. The mass signals were assigned to the following fragments: 964 [M + H − 2H_2_O]^+^, 954 [M + H − H_2_O − CO]^+^, 883 [M + H − Val − H_2_O]^+^, 865 [M + H − Val − 2H_2_O]^+^, 797 [M + 2H − (Asp + BA) − H_2_O]^+^, 779 [M + 2H − (Asp + BA) − 2H_2_O]^+^, 751 [M + 2H − (Asp + BA) − 2H_2_O − CO]^+^, 692 [M + H − (Val + MeHty) − H_2_O]^+^, 674 [M + H − (Val + MeHty) − 2H_2_O]^+^, 698 [M + 2H − Val − (Asp + BA) − H_2_O]^+^, 680 [M + 2H − Val − (Asp + BA) − 2H_2_O]^+^, 646 [M + H − (Val + MeHty) − 2H_2_O − CO]^+^, 434 [Ahp + Phe + MeHty + H − H_2_O]^+^, 432 [M + H − (Val + MeHty + Phe + Ahp) − H_2_O]^+^, 386 [BA + Asp + Thr + Val + H]^+^, 322 [Phe(−N) + MeHty + H]^+^, 269 [Asp + Thr + Val + H − H_2_O − CO]^+^, 243 [Ahp + Phe + H − H_2_O]^+^, 215 [Ahp + Phe + H − H_2_O − CO]^+^, 164 MeHty immonium ion, 136 Tyr immonium ion, 120 Phe immonium ion; Figure S12. Chemical structure (**A**) and product ion mass spectra of cyanopeptolin CP969 [Thr+Tyr+Ahp+Phe+MePhe+Val]Asp+BA with precursor ion [M + H − H_2_O]^+^ at *m*/*z* 952. The spectra were recorded with application of QTOF (**B**) and QTRAP (**C**) mass spectrometers. The mass signals were assigned to the following fragments: 934 [M + H − 2H_2_O]^+^, 924 [M + H − H_2_O − CO]^+^, 853 [M + H − Val − H_2_O]^+^, 835 [M + H − Val − 2H_2_O]^+^, 791 [M + H − MePhe − H_2_O]^+^, 767 [M + 2H − (Asp + BA) − H_2_O]^+^, 749 [M + 2H − (Asp + BA) − 2H_2_O]^+^, 692 [M + H − (Val + MePhe) − H_2_O]^+^, 674 [M + H − (Val + MePhe) − 2H_2_O]^+^, 432 [M + H − (Val + MePhe + Phe + Ahp) − H_2_O]^+^, 414 [M + H − (Val + MePhe + Phe + Ahp) − 2H_2_O]^+^, 404 [Ahp + Phe + MePhe + H − H_2_O]^+^, 386 [BA + Asp + Thr + Val + H]^+^, 297 [Asp + Thr + Val + H − H_2_O]^+^, 243 [Ahp + Phe + H − H_2_O]^+^, 215 [Ahp + Phe + H − H_2_O − CO]^+^, 134 MePhe immonium ion, 136 Tyr immonium ion, 120 Phe immonium ion; Figure S13. ^1^H-NMR Spectrum of cyanopeptolin CP985 in DMSO-d_6_; Figure S14. HSQC Spectrum of cyanopeptolin CP985 in DMSO-d_6_; Figure S15a. HMBC Spectrum of cyanopeptolin CP985 in DMSO-d_6_; Figure S15b. Detailed NH−C=O region of the HMBC spectrum of cyanopeptolin CP985; Figure S15c. Detailed aromatic region of the HMBC spectrum of cyanopeptolin CP985; Figure S16. COSY Spectrum of cyanopeptolin CP985 in DMSO-d_6_; Figure S17a. TOCSY Spectrum of cyanopeptolin CP985 in DMSO-d_6_; Figure S17b. Amino acid spin systems in the diagnostic region of the TOCSY spectrum of cyanopeptolin CP985; Figure S18a. ROESY Spectrum of cyanopeptolin CP985 in DMSO-d_6_; Figure S18b. Overlaid fragments of TOCSY (green) and ROESY (red) spectra of cyanopeptolin CP985; Figure S19. ^1^H-NMR Spectrum of cyanopeptolin CP962 in DMSO-d_6_; Figure S20. HSQC Spectrum of cyanopeptolin CP962 in DMSO-d_6_; Figure S21a. HMBC Spectrum of cyanopeptolin CP962 in DMSO-d_6_; Figure S21b. Detailed NH–C=O region of the HMBC spectrum of cyanopeptolin CP962; Figure S21c. Detailed aromatic region of the HMBC spectrum of cyanopeptolin CP962; Figure S22. COSY Spectrum of cyanopeptolin CP962 in DMSO-d_6_; Figure S23a. TOCSY Spectrum of cyanopeptolin CP962 in DMSO-d_6_; Figure S23b. Amino acid spin systems in the diagnostic region of the TOCSY spectrum of cyanopeptolin CP962; Figure S24a. ROESY Spectrum of cyanopeptolin CP962 in DMSO-d_6_; Figure S24b. Overlaid fragments of TOCSY (green) and ROESY (red) spectra of cyanopeptolin CP962.
