# Supplementary material for: Cyanopeptolins with Trypsin and Chymotrypsin Inhibitory Activity from the Cyanobacterium Nostoc edaphicum CCNP1411
Source: Mar Drugs. 2018 Jun 26;16(7):220. doi: 10.3390/md16070220 (PMC6070996; doi:10.3390/md16070220)
Supplement: Supplementary file 1 [file marinedrugs-16-00220-s001.pdf]

Supplementary Material: Cyanopeptolins with trypsin and chymotrypsin inhibitory activity from the cyanobacterium *Nostoc edaphicum* CCNP1411

Hanna Mazur-Marzec[1,2]*, Anna Fidor[1], Marta Cegłowska[2], Ewa Wieczerzak[3], Magdalena Kropidłowska[3], Marie Goua[4], Jenny Macaskill [4], Christine Edwards[4]

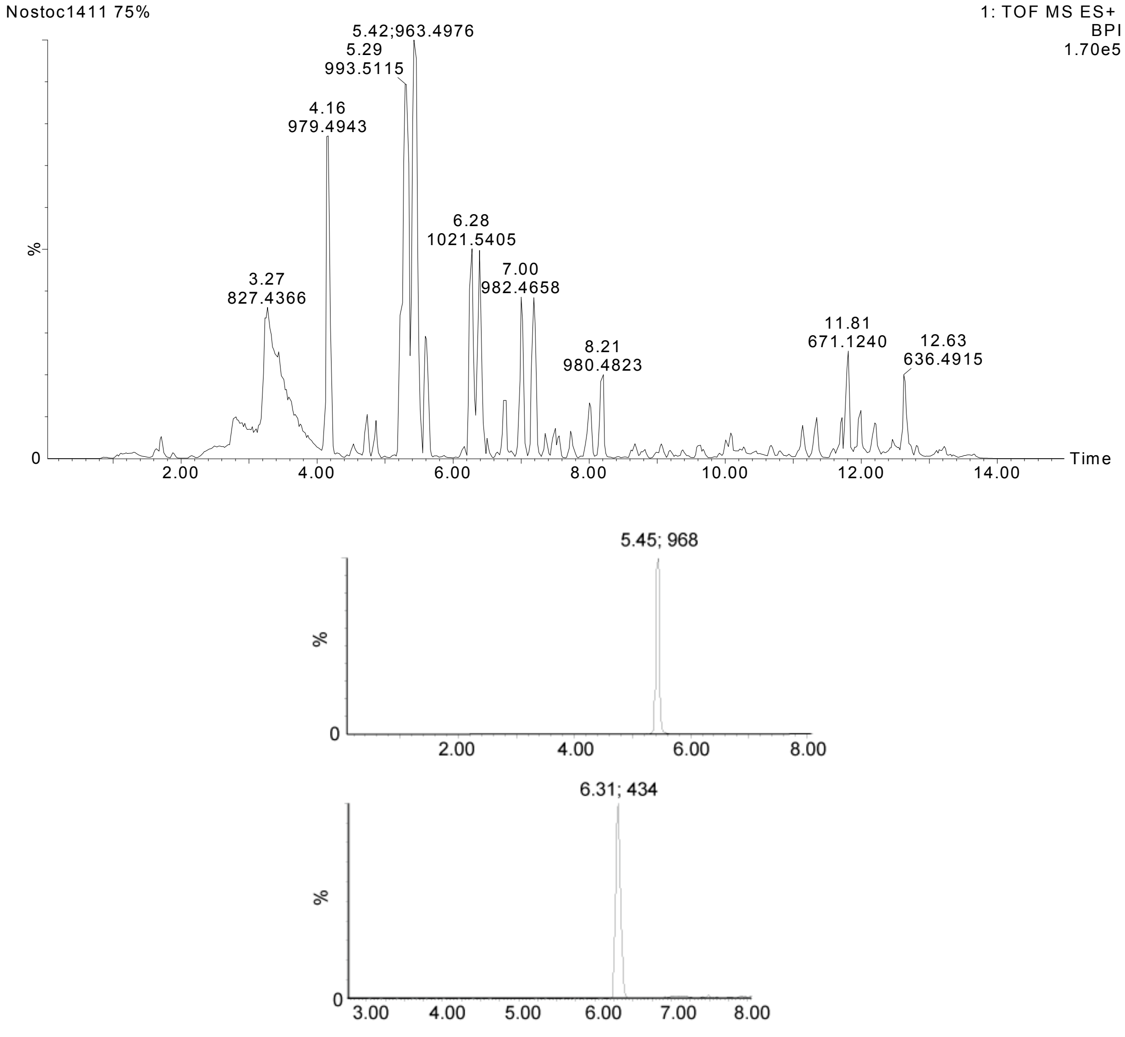

Figure S1. LC-MS/MS chromatogram of cyjanopeptolins (CPs) in crude extract from *Nostoc edaphicum* CCNP1411 (A) and chromatograms of isolated peptides: CP962 (B) and CP985 (C).

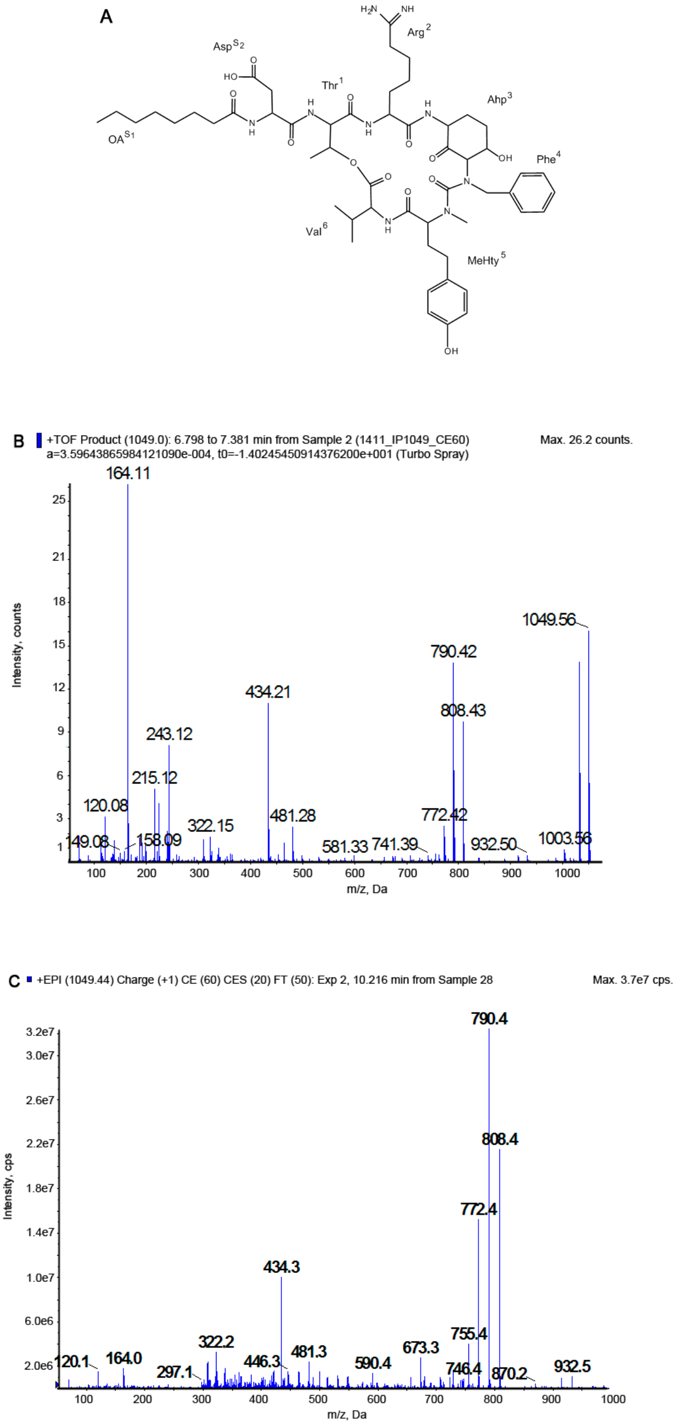

Figure S2. Chemical structure (**A**) and product ion mass spectra of cyanopeptolin CP1049 [Thr+Arg+Ahp+Phe+MeHty+Val]Asp+OA with precursor ion $[M + H]^+$ at *m/z* 1049. The spectra were recorded with application of a hybride quadrupole/time-of-flight mass spectrometer (QTOF) (**B**) and a hybride triple quadrupole/linear ion trap mass spectrometer (QTRAP) (**C**). The mass signals were assigned to the following fragments: 1031 $[M + H - H_2O]^+$, 1003 $[M + H - H_2O - CO]^+$, 932 $[M + H - Val - H_2O]^+$, 914 $[M + H - Val - 2H_2O]^+$, 808 $[M + 2H - (Asp + OA)]^+$, 790 $[M + 2H - (Asp + OA) - H_2O]^+$, 772 $[M + 2H - (Asp + OA) - 2H_2O]^+$, 741 $[M + H - (Val + MeHty) - H_2O]^+$, 673 $[M + 2H - Val - (Asp + OA) - 2H_2O]^+$, 481 $[OA+Asp + Thr + Arg + H - H_2O]^+$, 434 $[Ahp + Phe + MeHty + H - H_2O]^+$, 338 $[Arg + Thr + Val + H - H_2O]^+$, 322 $[Phe(-N) + MeHty + H]^+$, 297 $[Asp + Thr + Val + H - H_2O]^+$, 243 $[Ahp + Phe + H - H_2O]^+$, 215 $[Ahp + Phe + H - H_2O - CO]^+$, 164 MeHty immonium ion, 120 Phe immonium ion, 70-Arg.

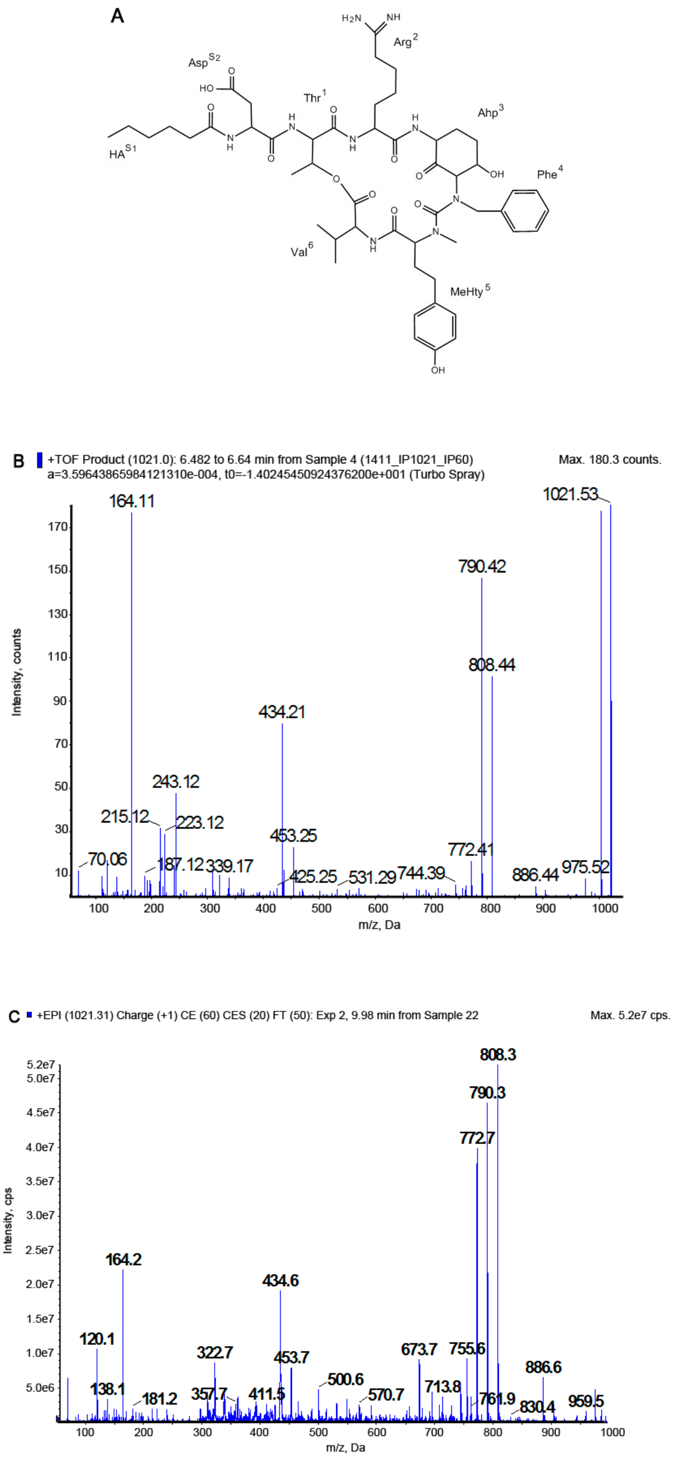

Figure S3. Chemical structure (A) and product ion mass spectra of cyanopeptolin CP1020 [Thr+Arg+Ahp+Phe+MeHty+Val]Asp+HA with precursor ion $[M+H]^+$ at *m/z* 1021. The spectra were recorded with application of QTOF (B) and QTRAP (C) mass spectrometers. The mass signals were assigned to the following fragments: 1003 $[M+H-H_2O]^+$, 975 $[M+H-H_2O-CO]^+$, 886 $[M+H-Val-2H_2O]^+$, 808 $[M+2H-(Asp+HA)]^+$, 790 $[M+2H-(Asp+HA)-H_2O]^+$, 772 $[M+2H-(Asp+OA)-2H_2O]^+$, 713 $[M+H-(Val+MeHty)-H_2O]^+$, 691 $[M+2H-Val-(Asp+HA)-H_2O]^+$, 673 $[M+2H-Val-(Asp+HA)-2H_2O]^+$, 453 $[HA+Asp+Thr+Arg+H-H_2O]^+$, 434 $[Ahp+Phe+MeHty+H-H_2O]^+$, 338 $[Arg+Thr+Val+H-H_2O]^+$, 322 $[Phe(-N)+MeHty+H]^+$, 297 $[Asp+Thr+Val+H-H_2O]^+$, 243 $[Ahp+Phe+H-H_2O]^+$, 215 [Ahp+Phe+H-H_2O-CO]+, 164 MeHty immonium ion, 120 Phe immonium ion, 70-Arg.

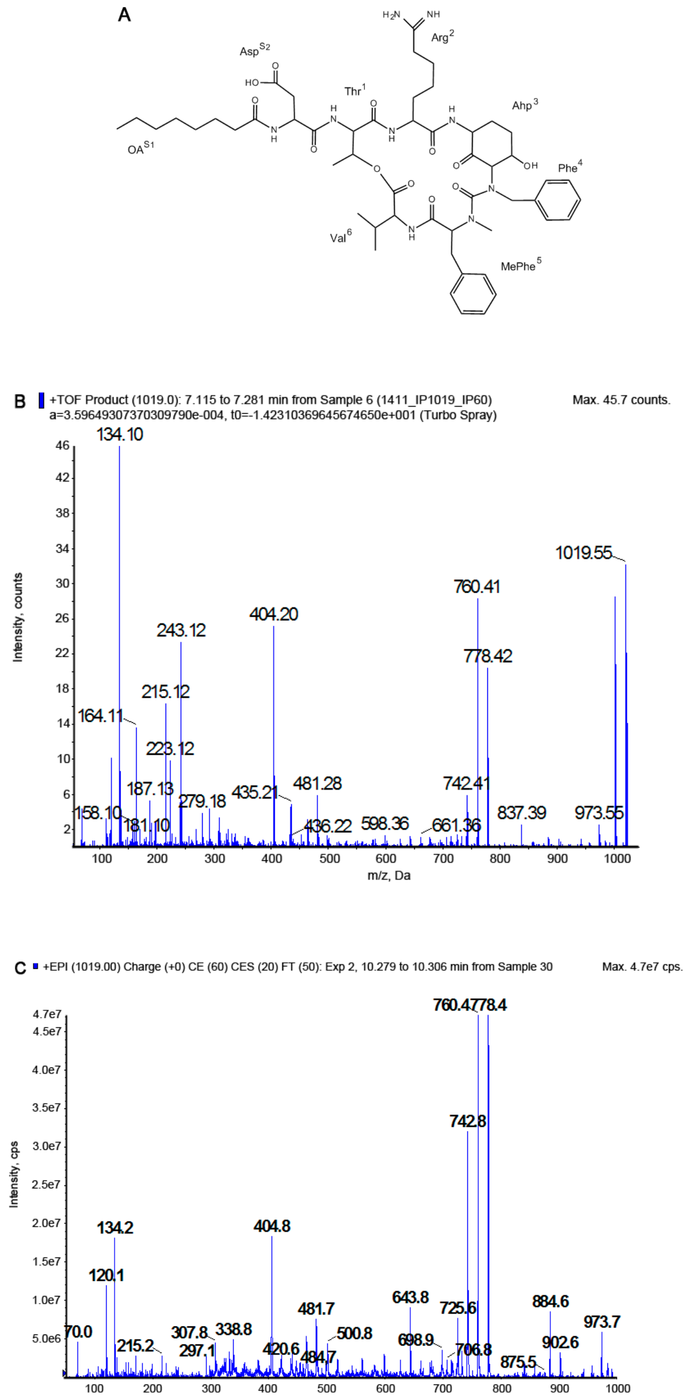

Figure S4. Chemical structure (**A**) and product ion mass spectra of cyanopeptolin CP1018 [Thr+Arg+Ahp+Phe+MePhe+Val]Asp+OA with precursor ion $[M+H]^+$ at *m/z* 1019. The spectra were recorded with application of QTOF (**B**) and QTRAP (**C**) mass spectrometers. The mass signals were assigned to the following fragments: 1001 $[M + H - H_2O]^+$, 983 $[M + H - 2H_2O]^+$, 973 $[M + H - H_2O - CO]^+$, 902 $[M + H - Val - H_2O]^+$, 884 $[M + H - Val - 2H_2O]^+$, 778 $[M + 2H - (Asp + OA)]^+$, 760 [M + 2H -– (Asp + OA) − $H_2O]^+$, 742 $[M + 2H - (Asp + OA) - 2H_2O]^+$, 661 $[M + 2H - Val - (Asp+OA) - H_2O]^+$, 643 $[M + 2H - Val - (Asp + OA) - 2H_2O]^+$, 481 $[OA + Asp + Thr + Arg + H - H_2O]^+$, 404 $[Ahp + Phe + MePhe + H - H_2O]^+$, 338 $[Arg + Thr + Val + H - H_2O]^+$, 308 $[Phe(-N) + MeTyr + H]^+$, 297 $[Asp + Thr + Val + H - H_2O]^+$, 243 $[Ahp + Phe + H - H_2O]^+$, 215 $[Ahp + Phe + H - H_2O - CO]^+$, 134 MePhe immonium ion, 120 Phe immonium ion, 70-Arg.

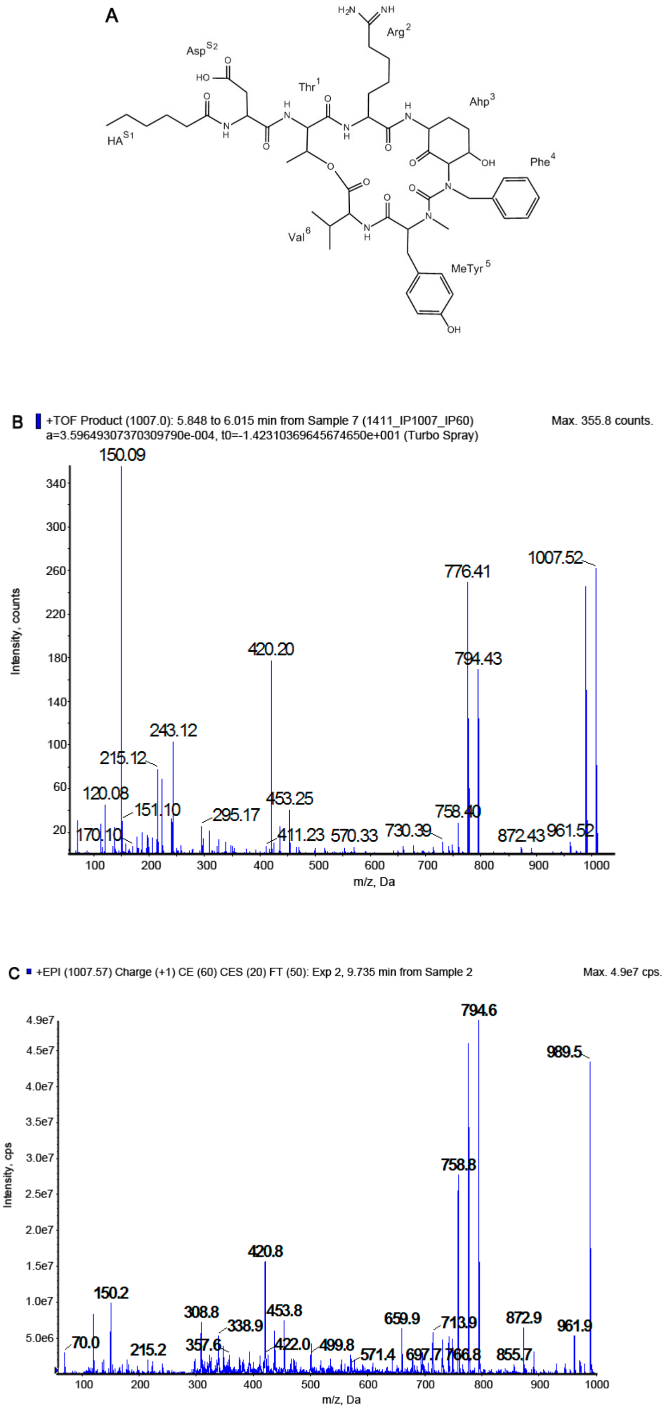

Figure S5. Chemical structure (A) and product ion mass spectra of cyanopeptolin CP1006 [Thr+Arg+Ahp+Phe+MeTyr+Val]Asp+HA with precursor ion [M + H]$^+$ at *m/z* 1007. The spectra were recorded with application of QTOF (B) and QTRAP (A) mass spectrometers. The mass signals were assigned to the following fragments: 989 [M + H − H_2O]$^+$, 961 [M + H − H_2O − CO]$^+$, 872 [M + H − Val − 2H_2O]$^+$, 794 [M + 2H − (Asp + HA)]$^+$, 776 [M + 2H − (Asp + HA) − H_2O]$^+$, 766 [M + 2H − (Asp + HA) − CO]$^+$, 758 [M + 2H − (Asp + HA) − 2H_2O]$^+$, 713 [M + H − (Val + MeHTyr) − H_2O]$^+$, 659 [M + 2H − Val − (Asp + HA) − 2H_2O]$^+$, 453 [HA + Asp + Thr + Arg + H − H_2O]$^+$, 420 [Ahp + Phe + MeTyr + H − H_2O]$^+$, 338 [Arg + Thr + Val + H − H_2O]$^+$, 308 [Phe(−N) + MeTyr + H]$^+$, 297 [Asp + Thr + Val + H − H_2O]$^+$, 243 [Ahp + Phe + H − H_2O]$^+$, 215 [Ahp + Phe + H − H_2O − CO]$^+$, 150 MeTyr immonium ion, 120 Phe immonium ion, 70-Arg.

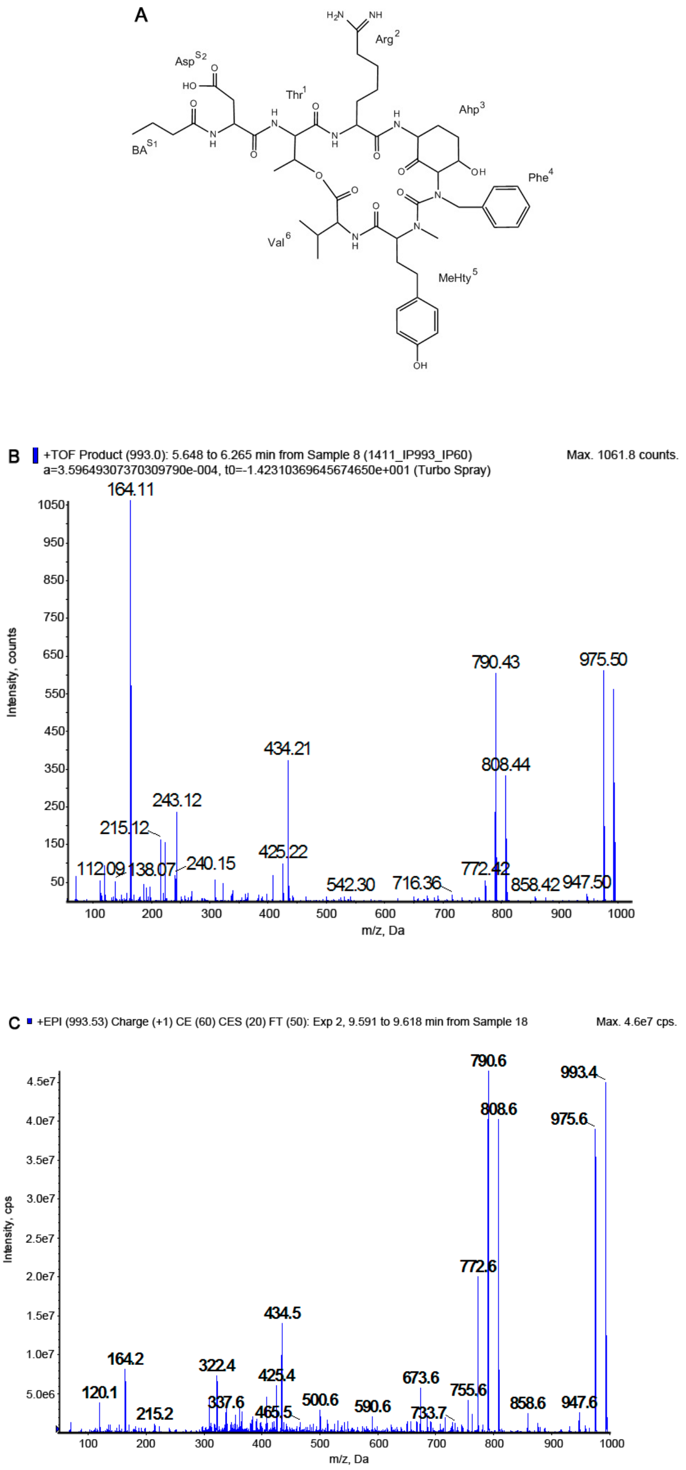

Figure S6. Chemical structure (A) and product ion mass spectra of cyanopeptolin CP992 [Thr+Arg+Ahp+Phe+MeHty+Val]Asp+BA with precursor ion [M + H]+ at m/z 993. The spectra were recorded with application of QTOF (B) and QTRAP (C) mass spectrometers. The mass signals were assigned to the following fragments: 975 [M + H − H2O]+, 947 [M + H − H2O − CO]+, 858 [M + H − Val −-2H2O]+, 808 [M + 2H − (Asp + BA)]+, 790 [M + 2H − (Asp + BA) − H2O]+, 772 [M + 2H − (Asp + BA) − 2H2O]+, 673 [M + 2H − Val − (Asp + BA) − 2H2O]+, 434 [Ahp + Phe + MeHty + H − H2O]+, 425 [BA + Asp + Thr + Arg + H − H2O]+, 338 [Arg + Thr + Val + H − H2O]+, 322 [Phe(−N) + MeHty + H]+, 243 [Ahp + Ph + H − H2O]+, 215 [Ahp + Phe + H − H2O − CO]+, 164 MeHty immonium ion, 120 Phe immonium ion, 70-Arg.

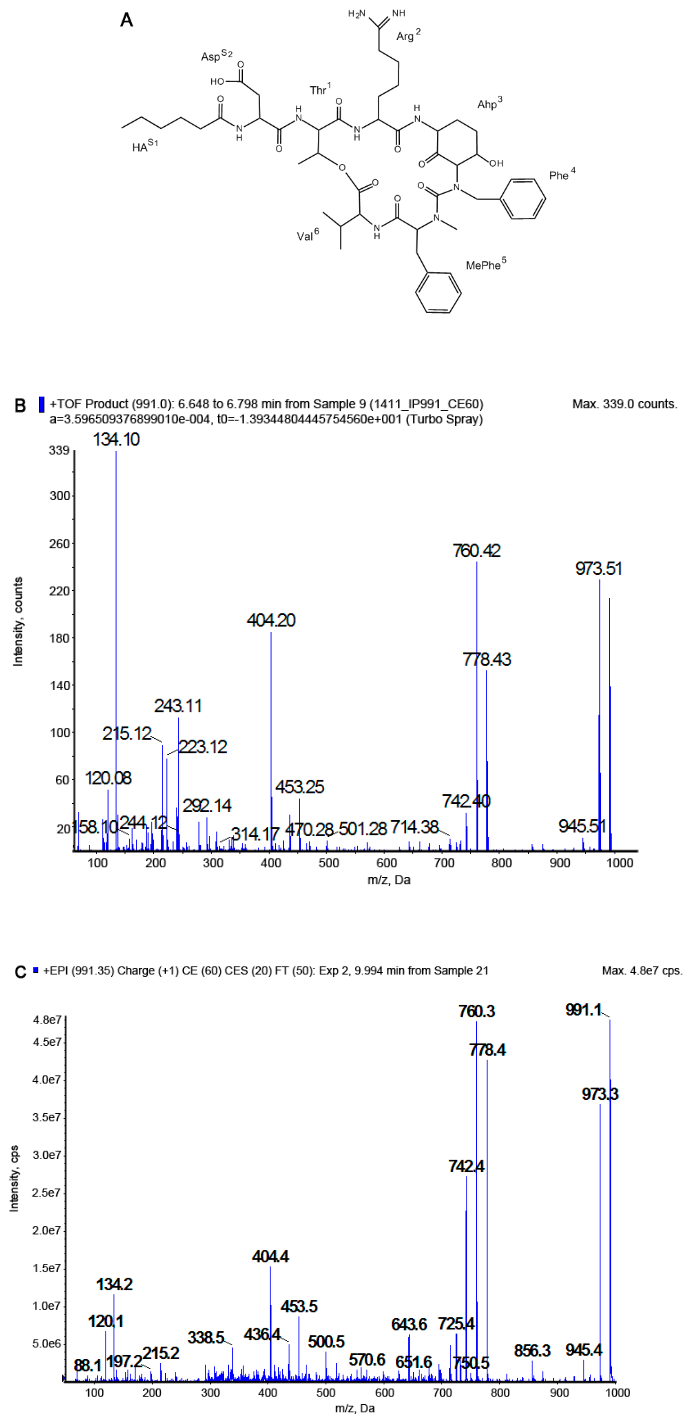

Figure S7. Chemical structure (**A**) and product ion mass spectra of cyanopeptolin CP990 [Thr+Arg+Ahp+Phe +MePhe+Val]Asp+HA with precursor ion $[M + H]^+$ at *m/z* 991. The spectra were recorded with application of QTOF (**B**) and QTRAP (**C**) mass spectrometers. The mass signals were assigned to the following fragments: 973 $[M + H - H_2O]^+$, 945 $[M + H - H_2O - CO]^+$, 856 $[M + H - Val - 2H_2O]^+$, 778 $[M + 2H - (Asp + HA)]^+$, 760 $[M + 2H - (Asp + HA) - H_2O]^+$, 750 $[M + 2H - (Asp + HA) - CO]^+$, 742 $[M + 2H\text{-}(Asp + HA) - 2H_2O]^+$, 643 $[M + 2H - Val - (Asp + HA) - 2H_2O]^+$, 453 $[HA + Asp + Thr + Arg + H - H_2O]^+$, 404 $[Ahp + Phe + MePhe + H - H_2O]^+$, 338 $[Arg + Thr + Val + H - H_2O]^+$, 297 $[Asp + Thr + Val + H - H_2O]^+$, 243 $[Ahp\text{+}Phe\text{+}H\text{-}H_2O]^+$, 215 $[Ahp\text{+}Phe\text{+}H\text{-}H_2O\text{-}CO]^+$, 134 MePhe immonium ion, 120 Phe immonium ion, 70-Arg.

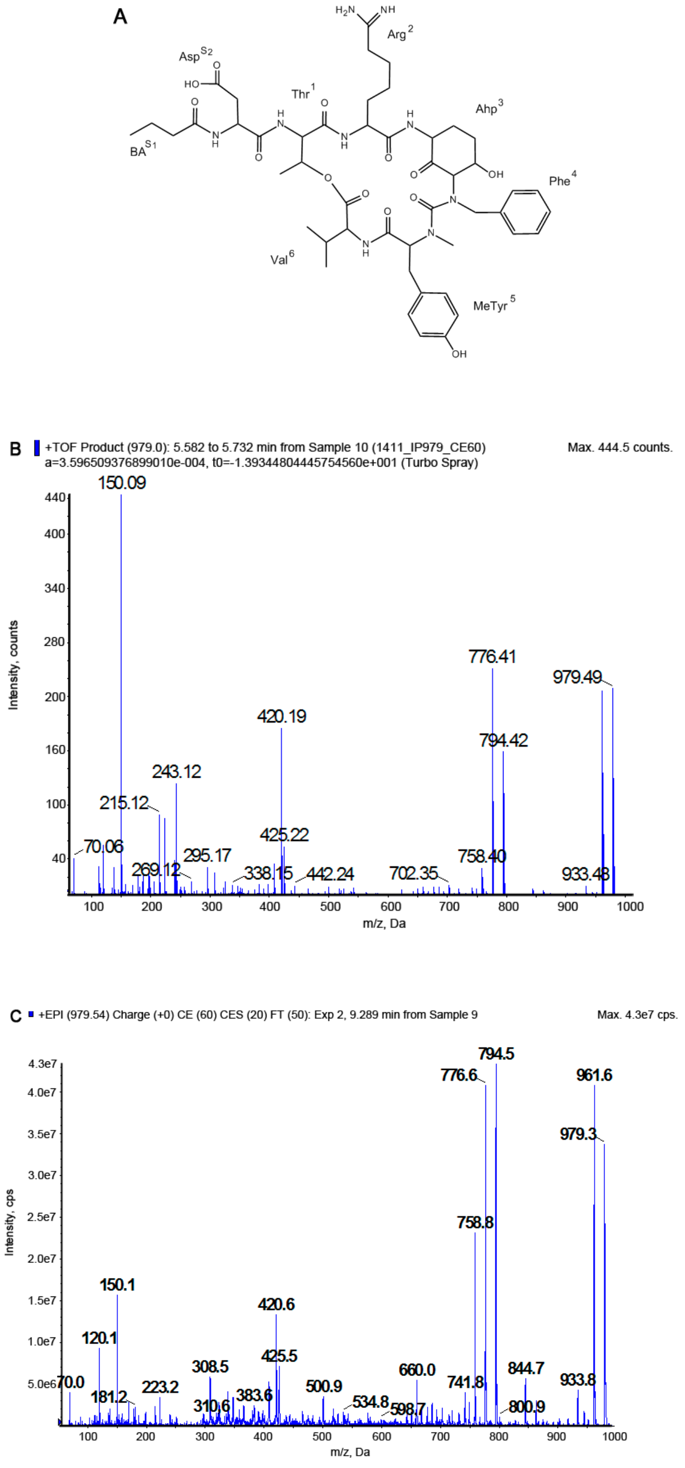

Figure S8. Chemical structure (**A**) and product ion mass spectra of cyanopeptolin CP978 [Thr+Arg+Ahp+Phe+MeTyr+Val]Asp+BA with precursor ion $[M+H]^+$ at *m/z* 979. The spectra were recorded with application of QTOF (**B**) and QTRAP (**C**) mass spectrometers. The mass signals were assigned to the following fragments: 961 $[M + H - H_2O]^+$, 933 $[M + H - H_2O - CO]^+$, 844 $[M + H - Val - 2H_2O]^+$, 794 $[M + 2H - (Asp + BA)]^+$, 776 $[M + 2H - (Asp + BA) - H_2O]^+$, 758 $[M + 2H - (Asp + BA) - 2H_2O]^+$, 659 $[M + 2H - Val - (Asp + BA) - 2H_2O]^+$, 425 $[BA + Asp + Thr + Arg + H - H_2O]^+$, 420 $[Ahp + Phe + MeTyr + H - H_2O]^+$, 338 $[Arg + Thr + Val + H - H_2O]^+$, 308 $[Phe(-N) + MeTyr + H]^+$, 243 $[Ahp + Phe + H - H_2O]^+$, 215 $[Ahp + Phe + H - H_2O - CO]^+$, 150 MeTyr immonium ion, 120 Phe immonium ion, 70-Arg.

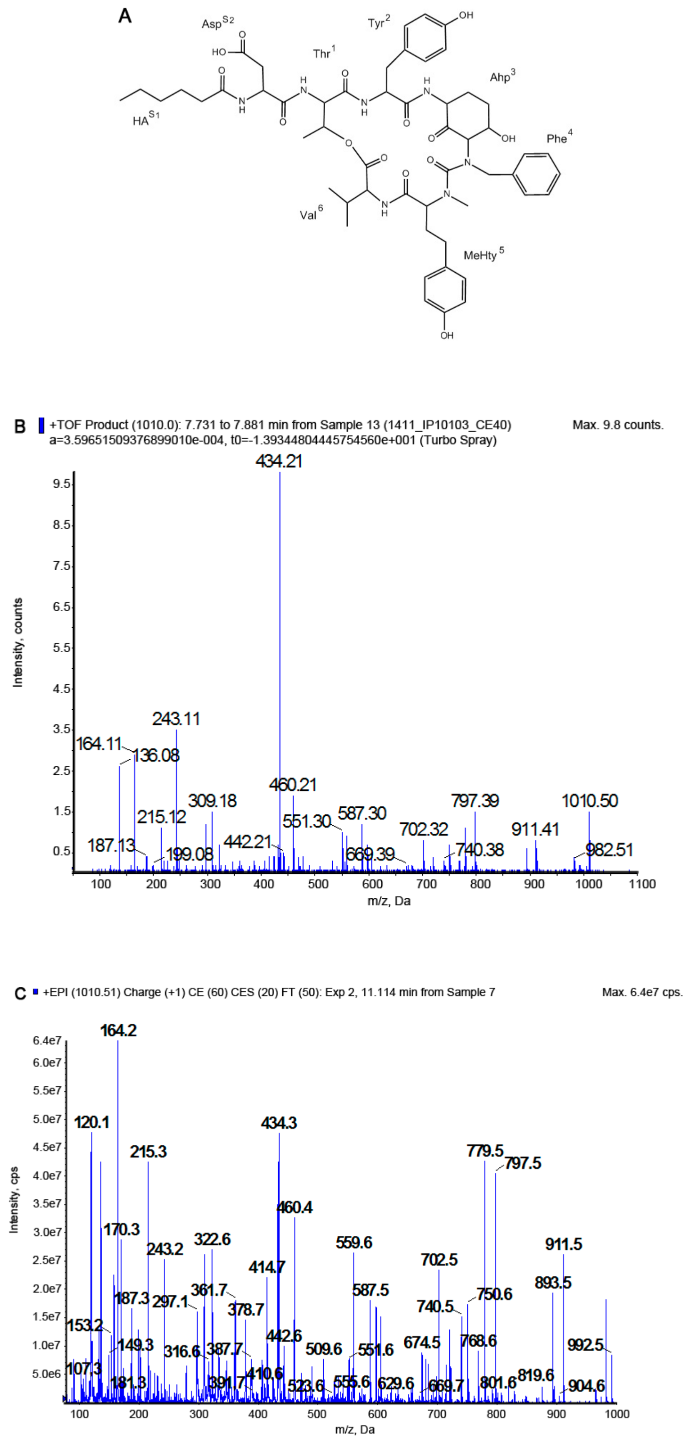

Figure S9. Chemical structure (**A**) and product ion mass spectra of cyanopeptolin CP1027 [Thr+Tyr+Ahp+Phe+MeHty+Val]Asp+HA with precursor ion $[M{+}H{-}H_2O]^+$ at *m/z* 1010. The spectra were recorded with application of QTOF (**B**) and QTRAP (**C**) mass spectrometers. The mass signals were assigned to the following fragments: 992 $[M + H - 2H_2O]^+$, 982 $[M + H - H_2O - CO]^+$, 964 $[M + H - 2H_2O - CO]^+$, 911 $[M + H - Val - H_2O]^+$, 893 $[M + H - Val - 2H_2O]^+$, 819 $[M + H - MeHty - H_2O]^+$, 797 $[M + 2H - (Asp + HA) - H_2O]^+$, 779 $[M + 2H - (Asp + HA) - 2H_2O]^+$, 751 $[M + 2H - (Asp + HA) - 2H_2O - CO]^+$, 702$[M + H - (Val + MeHty) - H_2O]^+$, 674 $[M + H - (Val + MeHty) - H_2O - CO]^+$, 460 $[M + H - (Val + MeHty + Phe + Ahp) - H_2O]^+$, 442 $[M + H - (Val + MeHty + Phe + Ahp) - 2H_2O]^+$, 434 $[Ahp + Phe + MeHty +H - H_2O]^+$, 322 $[Phe(-N) + MeHty + H]^+$, 297 $[Asp + Thr+ Val +H - H_2O]^+$, 243 $[Ahp +Phe + H - H_2O]^+$, 215 $[Ahp + Phe + H - H_2O -- CO]^+$, 164 MeHty immonium ion, 136 Tyr immonium ion, 120 Phe immonium ion.

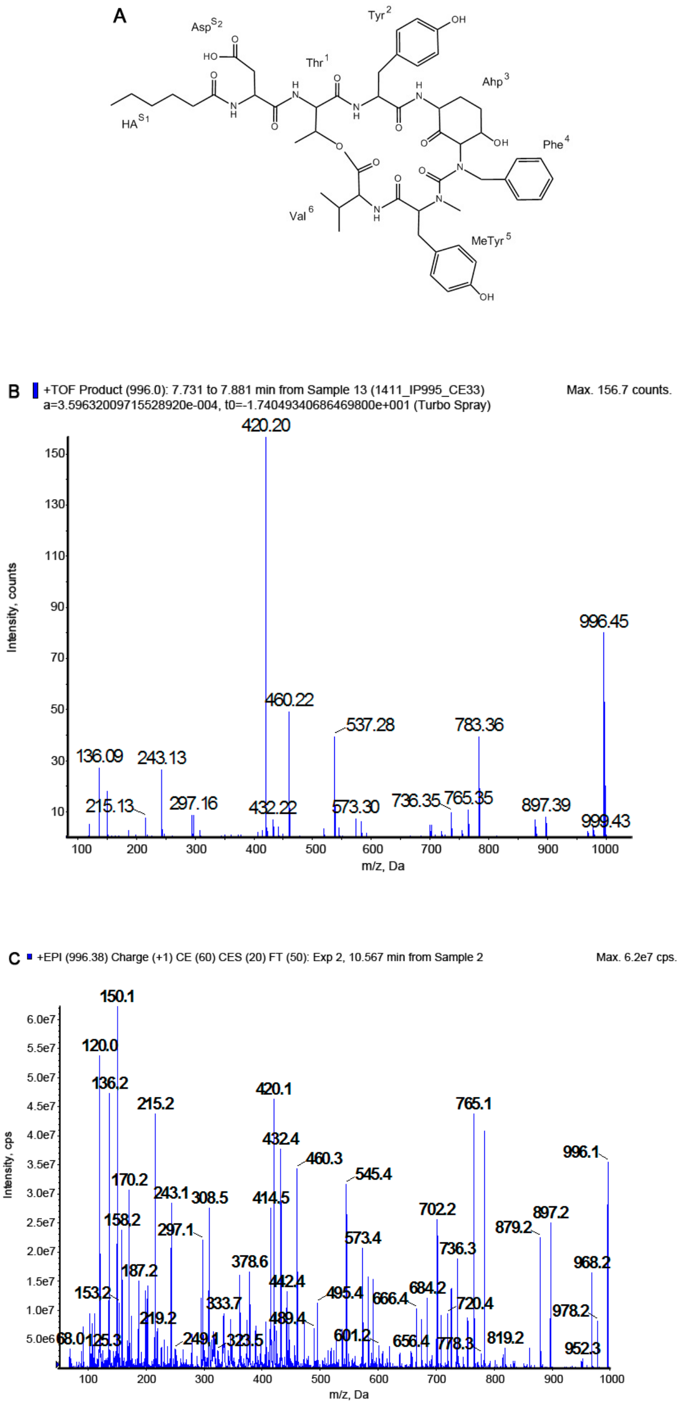

Figure S10. Chemical structure (**A**) and product ion mass spectra of cyanopeptolin CP1013 [Thr+Tyr+Ahp+Phe+MeTyr+Val]Asp+HA with precursor ion $[M+H-H_2O]^+$ at *m/z* 996. The spectra were recorded with application of QTOF (**B**) and QTRAP (**C**) mass spectrometers. The mass signals were assigned to the following fragments: 978 $[M + H - 2H_2O]^+$, 968 $[M + H - H_2O - CO]^+$, 897 $[M + H - Val - H_2O]^+$, 879 $[M + H - Val - 2H_2O]^+$, 819 $[M + H - MeTyr - H_2O]^+$, 783 $[M + 2H - (Asp + HA) - H_2O]^+$, 765 $[M + 2H - (Asp + HA) - 2H_2O]^+$, 736 $[M + H - (Asp + HA) - 2H_2O - CO]^+$, 720 $[M + H - (Val + MeTyr) - H_2O]^+$, 702 $[M + H - (Val + MeTyr) - 2H_2O]^+$, 666 $[M + 2H - Val - (Asp + HA) - 2H_2O]^+$, 460 $[M + H - (Val + MeTyr + Phe + Ahp) - H_2O]^+$, 420 $[Ahp + Phe + MeTyr + H - H_2O]^+$, 432 $[M + H - (Val + MeTyr + Phe + Ahp) - H_2O - CO]^+$, 414 $[HA + Asp + Thr + Val + H]^+$, 297 $[Asp + Thr + Val + H - H_2O]^+$, 243 $[Ahp + Phe + H - H_2O]^+$, 215 $[Ahp + Phe+ H - H_2O - CO]^+$, 150 MeTyr immonium ion, 136 Tyr immonium ion, 120 Phe immonium ion.

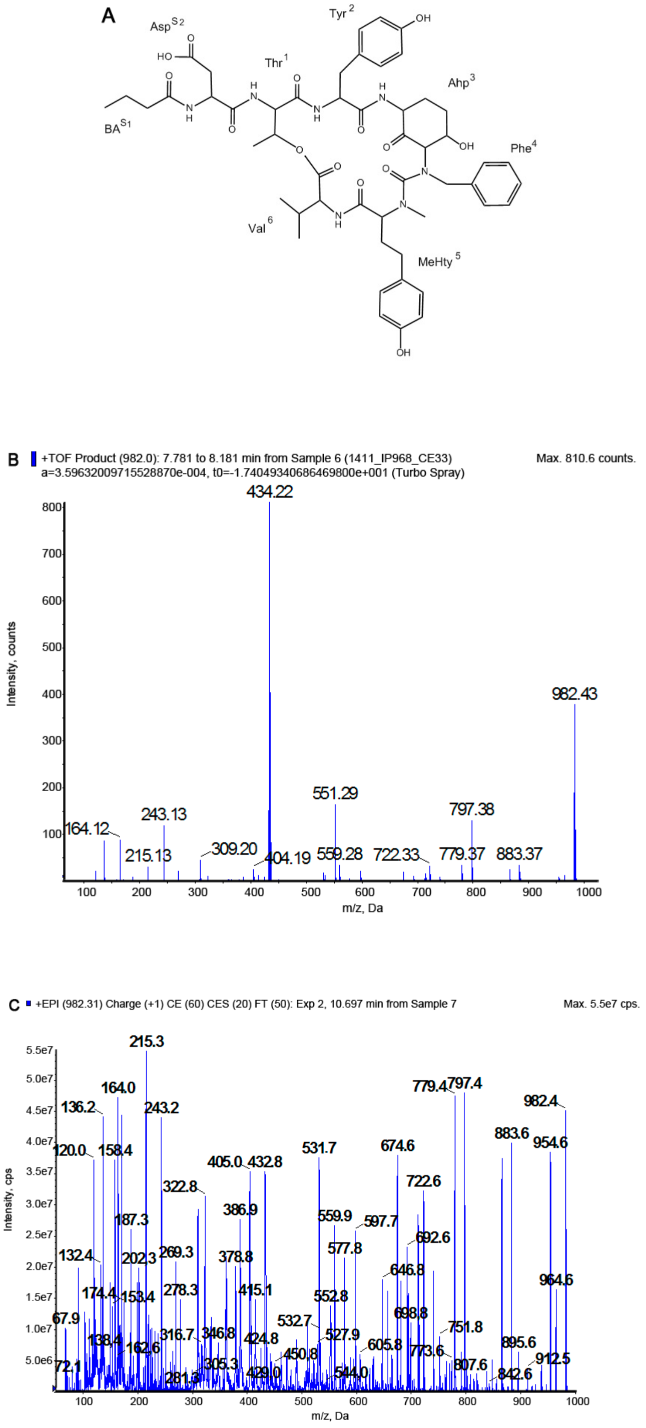

Figure S11. Chemical structure (**A**) and product ion mass spectra of cyanopeptolin CP999 [Thr+Tyr+Ahp+Phe+MeHty+Val]Asp+BA with precursor ion $[M+H-H_2O]^+$ at *m/z* 982. The spectra were recorded with application of QTOF (**B**) and QTRAP (**C**) mass spectrometers. The mass signals were assigned to the following fragments: 964 $[M + H - 2H_2O]^+$, 954 $[M + H - H_2O - CO]^+$, 883 $[M + H - Val - H_2O]^+$, 865 $[M + H - Val - 2H_2O]^+$, 797 $[M + 2H - (Asp + BA) - H_2O]^+$, 779 $[M + 2H - (Asp + BA) - 2H_2O]^+$, 751 $[M + 2H - (Asp + BA) - 2H_2O - CO]^+$, 692 $[M + H - (Val + MeHty) - H_2O]^+$, 674 $[M + H - (Val + MeHty) - 2H_2O]^+$, 698 $[M + 2H - Val - (Asp + BA) - H_2O]^+$, 680 $[M + 2H - Val - (Asp + BA) - 2H_2O]^+$, 646 $[M + H - (Val + MeHty) - 2H_2O - CO]^+$, 434 $[Ahp + Phe + MeHty + H - H_2O]^+$, 432 $[M + H - (Val + MeHty + Phe + Ahp) - H_2O]^+$, 386 $[BA + Asp + Thr + Val + H]^+$, 322 $[Phe(-N) + MeHty + H]^+$, 269 $[Asp + Thr + Val + H - H_2O - CO]^+$, 243 [Ahp + Phe + H −

$H_2O]^+$, 215 [Ahp + Phe + H − H_2O − CO]$^+$, 164 MeHty immonium ion, 136 Tyr immonium ion, 120 Phe immonium ion.

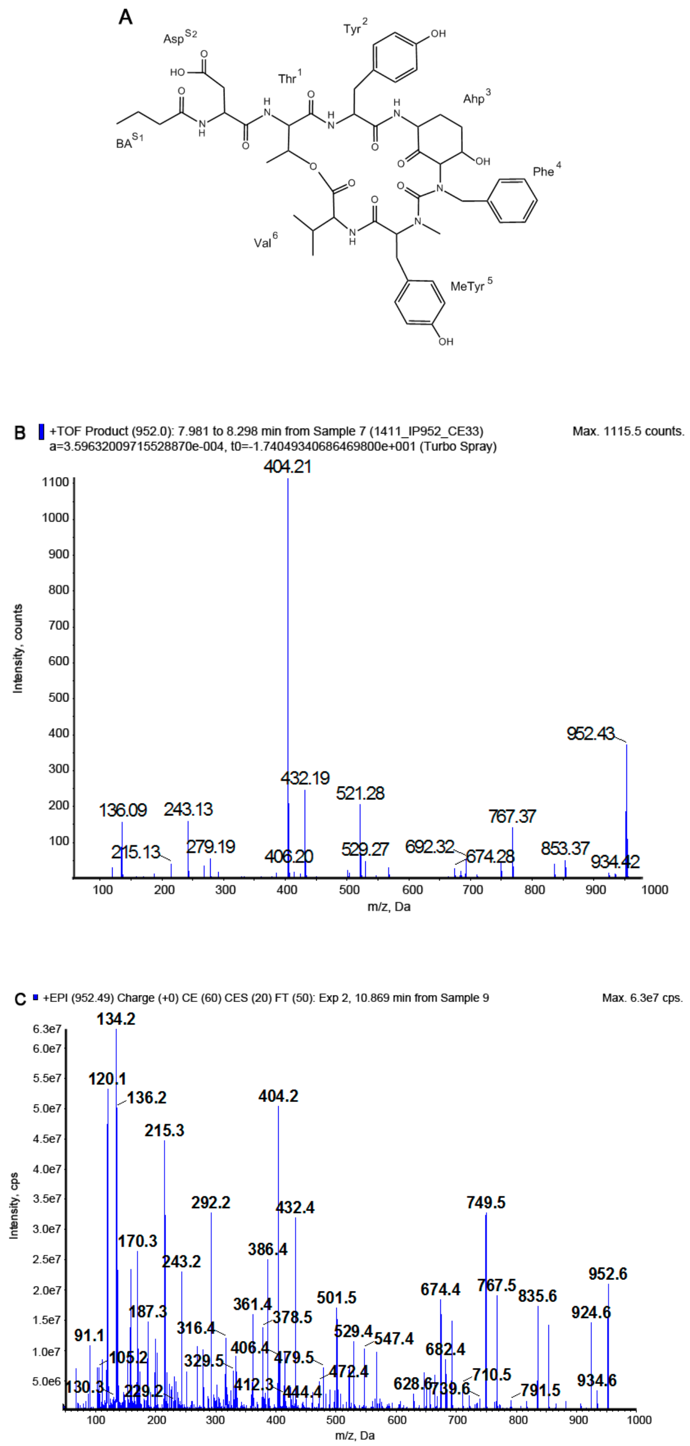

Figure S12. Chemical structure (**A**) and product ion mass spectra of cyanopeptolin CP969 [Thr+Tyr+Ahp+Phe+MePhe+Val]Asp+BA with precursor ion [M + H − $H_2O]^+$ at *m/z* 952. The spectra were recorded with application of QTOF (**B**) and QTRAP (**C**) mass spectrometers. The mass signals were assigned to the following fragments: 934 [M + H − $2H_2O]^+$, 924 [M + H − H_2O − CO]$^+$, 853 [M + H − Val − $H_2O]^+$, 835 [M + H − Val − $2H_2O]^+$, 791 [M + H − MePhe − $H_2O]^+$, 767 [M + 2H − (Asp + BA) − $H_2O]^+$, 749 [M + 2H − (Asp + BA) − $2H_2O]^+$, 692 [M + H − (Val + MePhe) − $H_2O]^+$, 674 [M + H − (Val + MePhe) − $2H_2O]^+$, 432 [M + H − (Val + MePhe + Phe + Ahp) − $H_2O]^+$, 414 [M + H − (Val + MePhe + Phe + Ahp) − $2H_2O]^+$, 404 [Ahp + Phe + MePhe + H − $H_2O]^+$,

386 [BA + Asp + Thr + Val + H]$^+$, 297 [Asp + Thr + Val + H − H_2O]$^+$, 243 [Ahp + Phe + H − H_2O]$^+$, 215 [Ahp + Phe + H − H_2O − CO]$^+$, 134 MePhe immonium ion, 136 Tyr immonium ion, 120 Phe immonium ion.

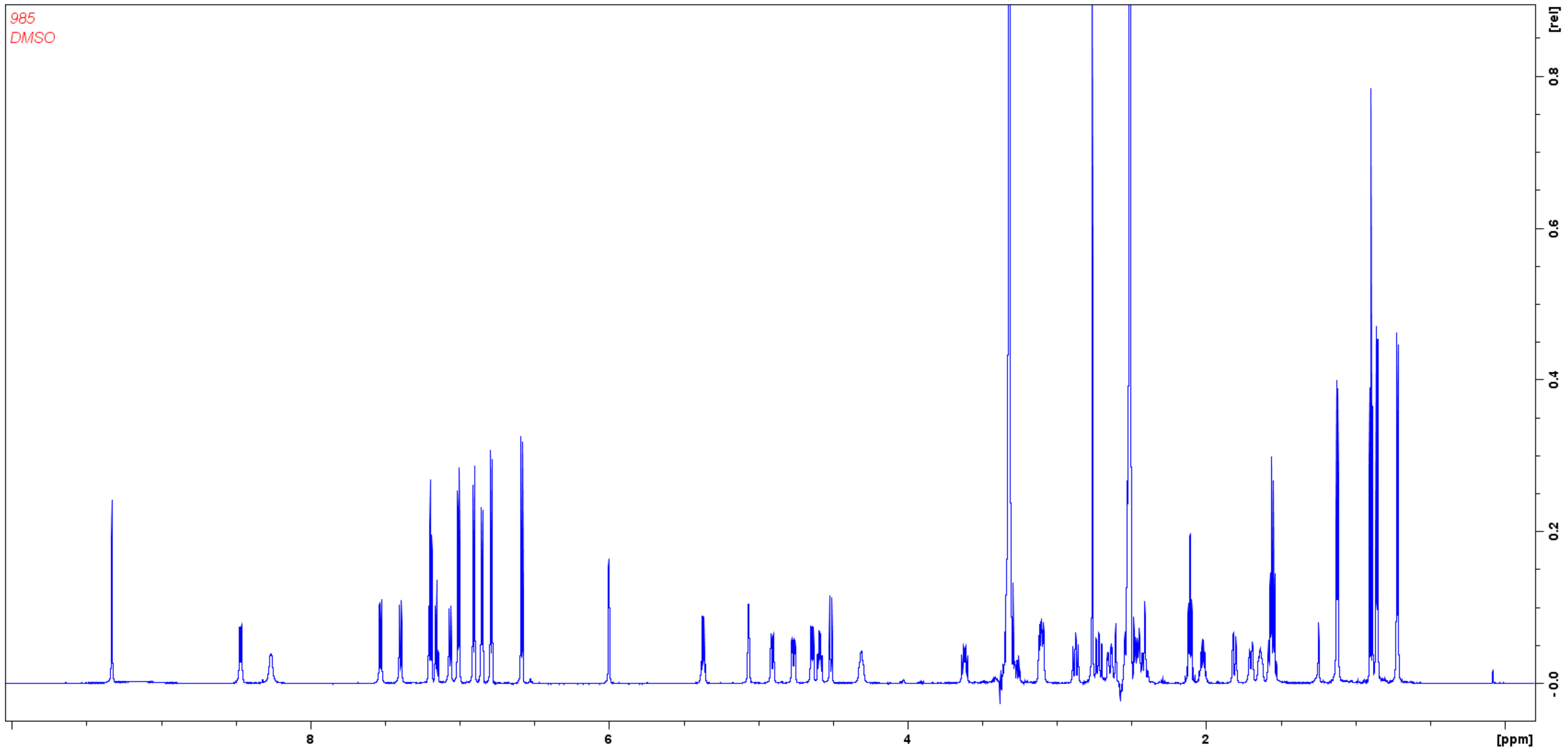

Figure S13. ¹H NMR Spectrum of cyanopeptolin CP985 in DMSO-d6.

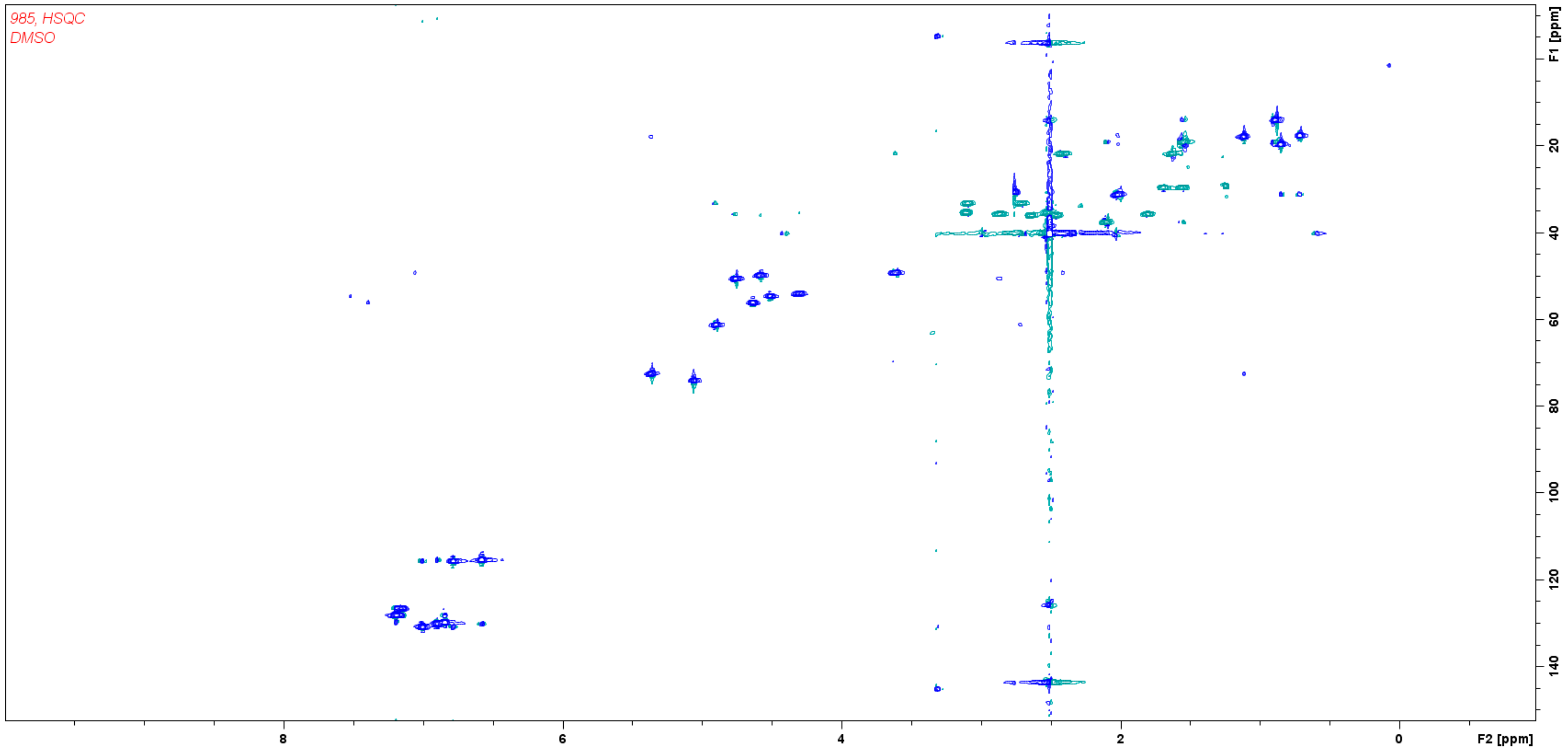

Figure S14. HSQC Spectrum of cyanopeptolin CP985 in DMSO-d6.

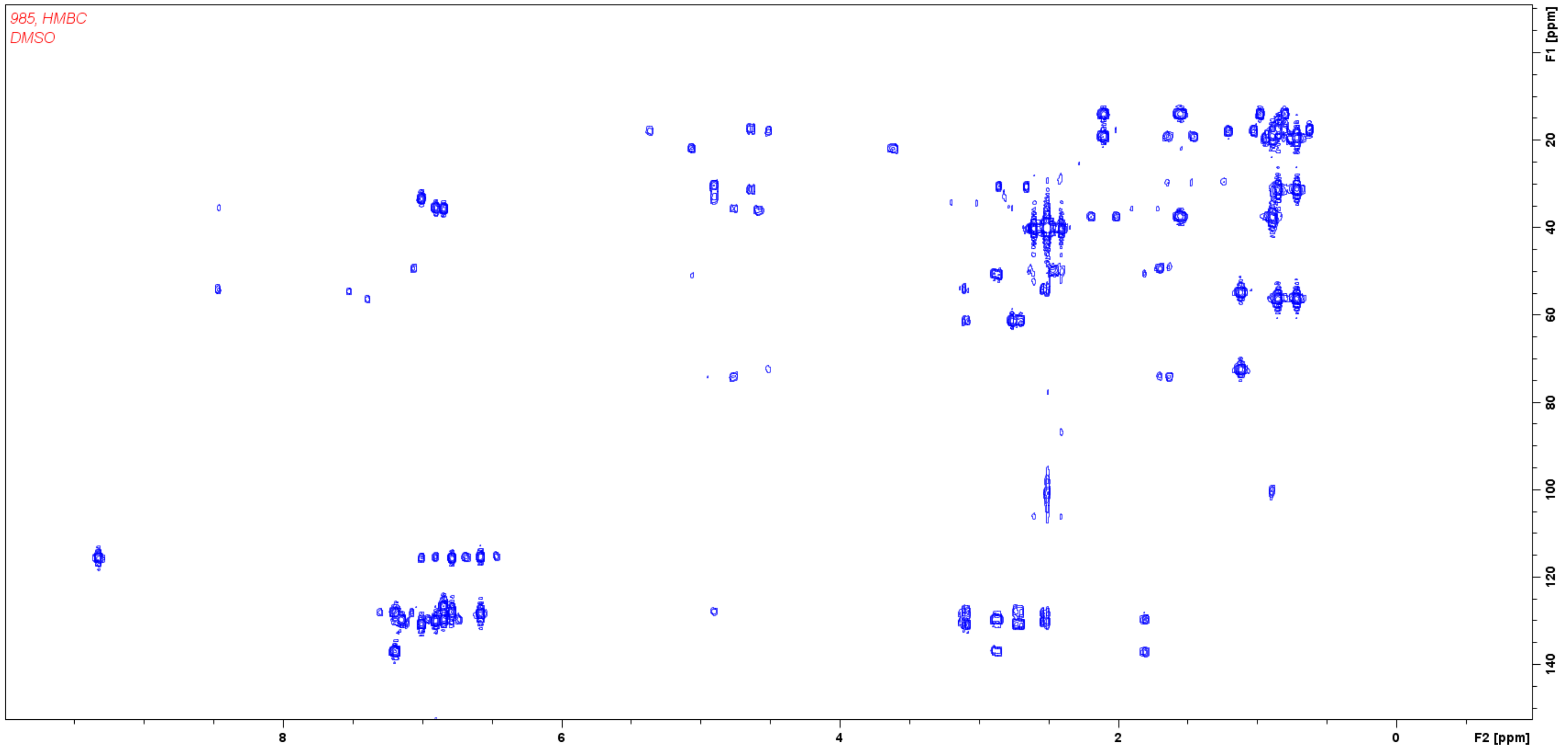

Figure S15a. HMBC Spectrum of cyanopeptolin CP985 in DMSO-d6.

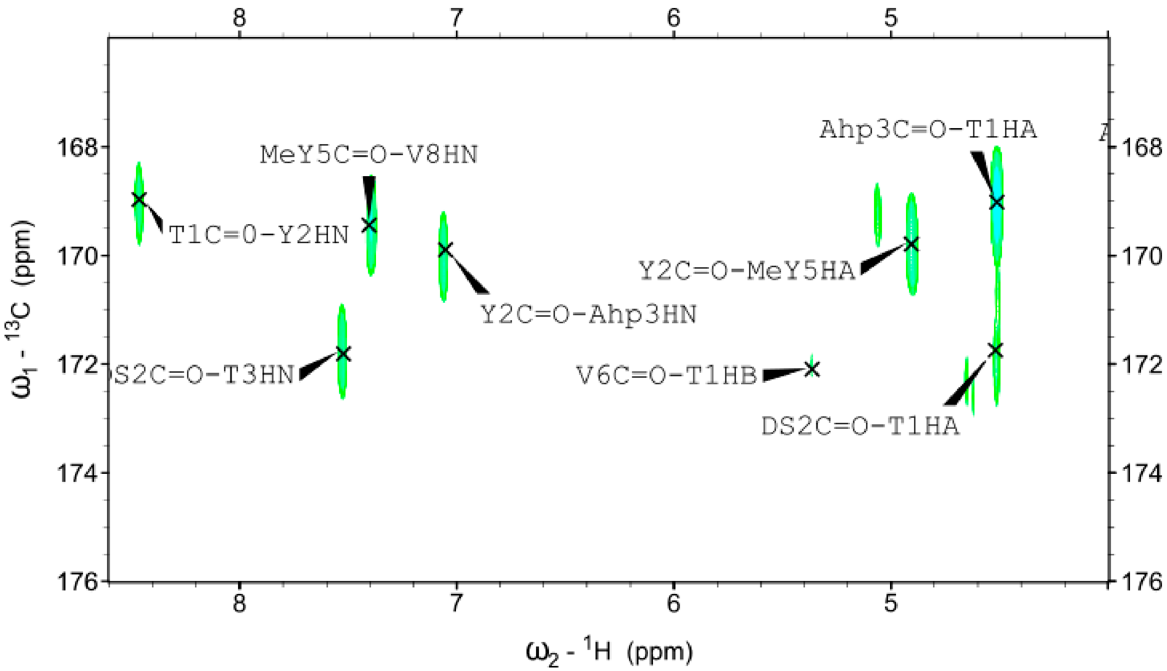

Figure S15b. Detailed NH – C=O region of the HMBC spectrum of cyanopeptolin CP985.

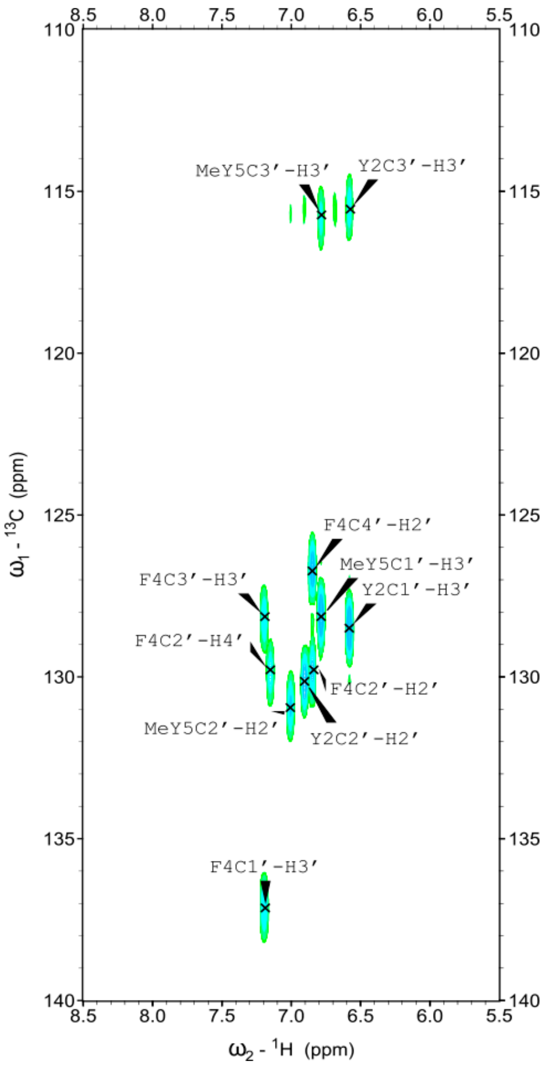

Figure S15c. Detailed aromatic region of the HMBC spectrum of cyanopeptolin CP985.

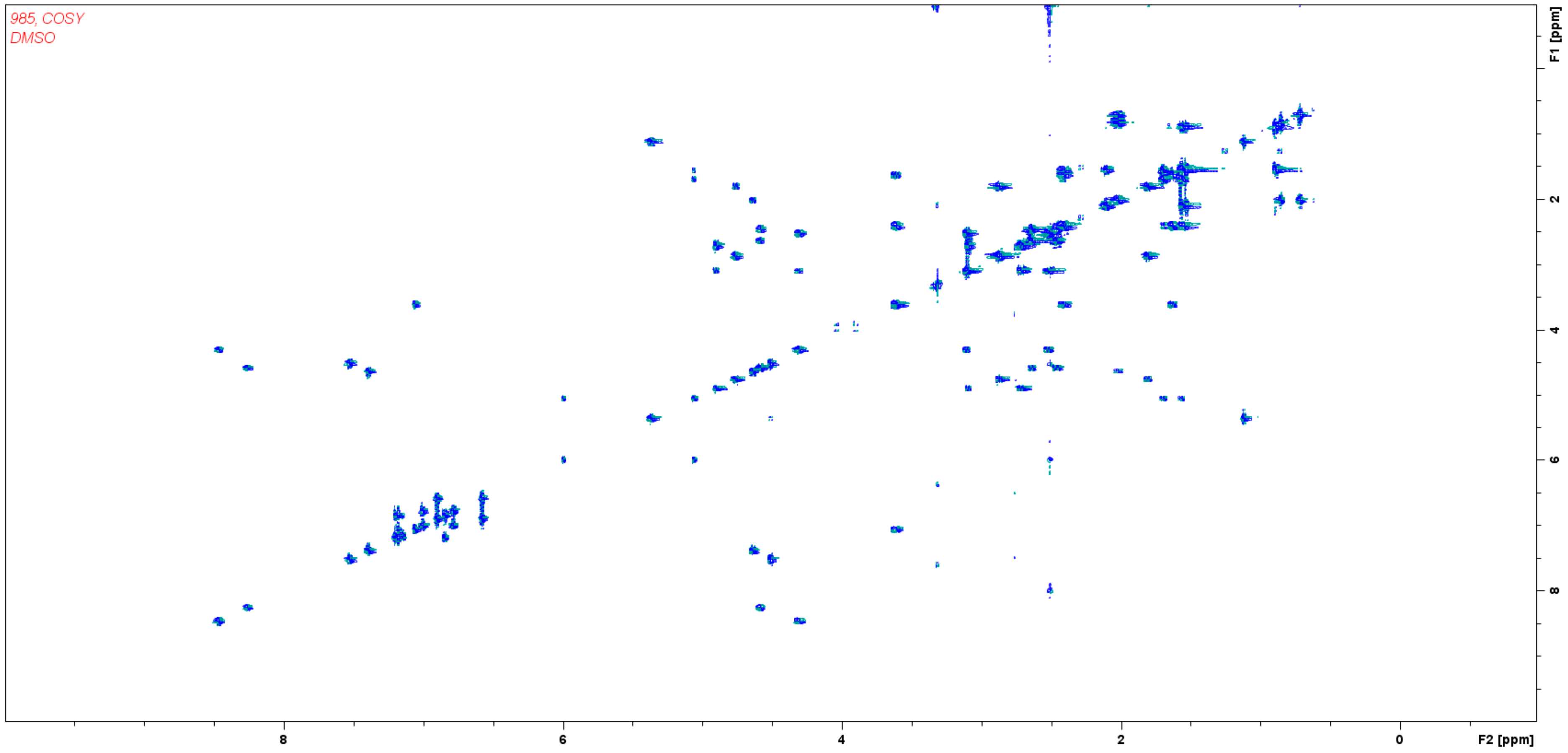

Figure S16. COSY Spectrum of cyanopeptolin CP985 in DMSO-d6.

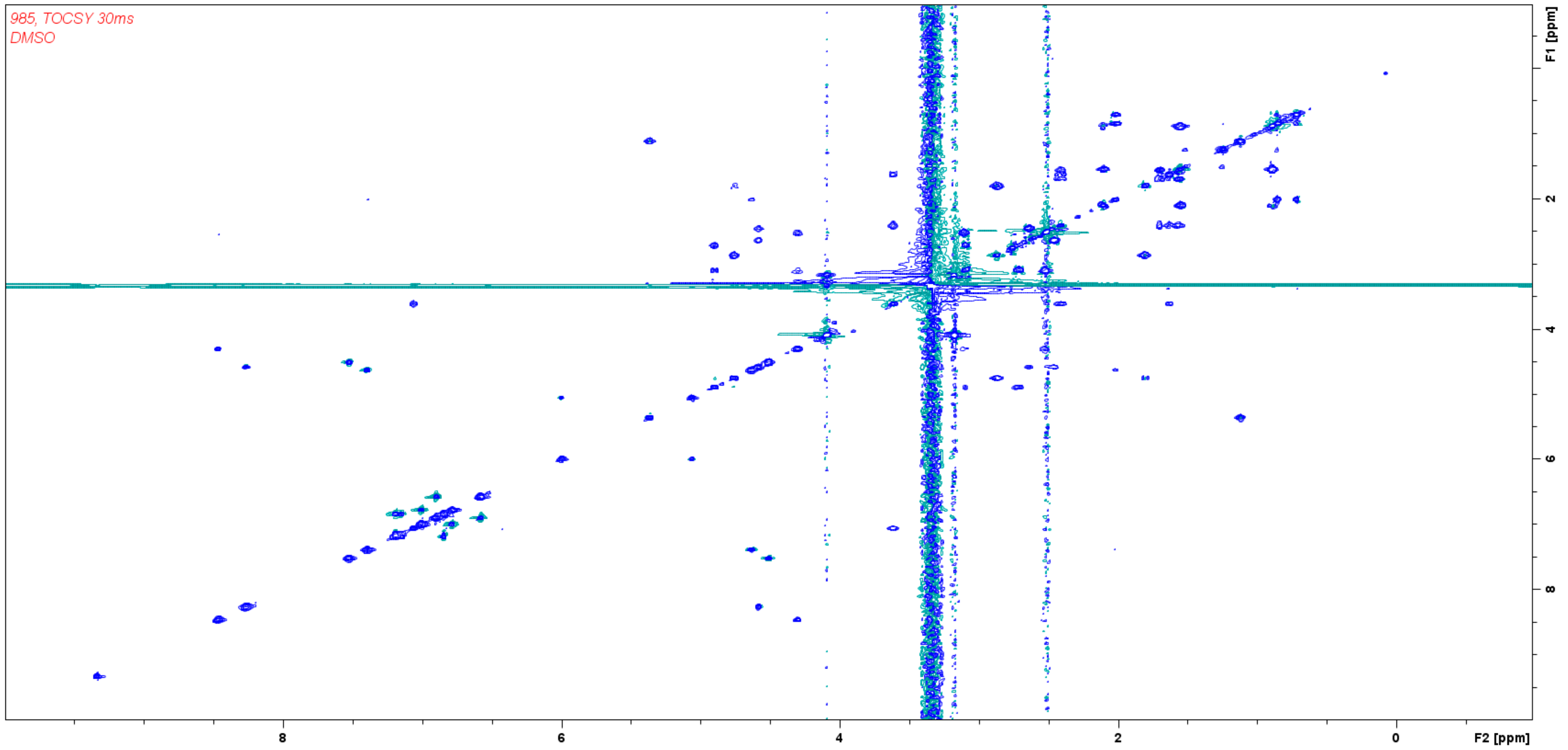

Figure S17a. TOCSY Spectrum of cyanopeptolin CP985 in DMSO-d6.

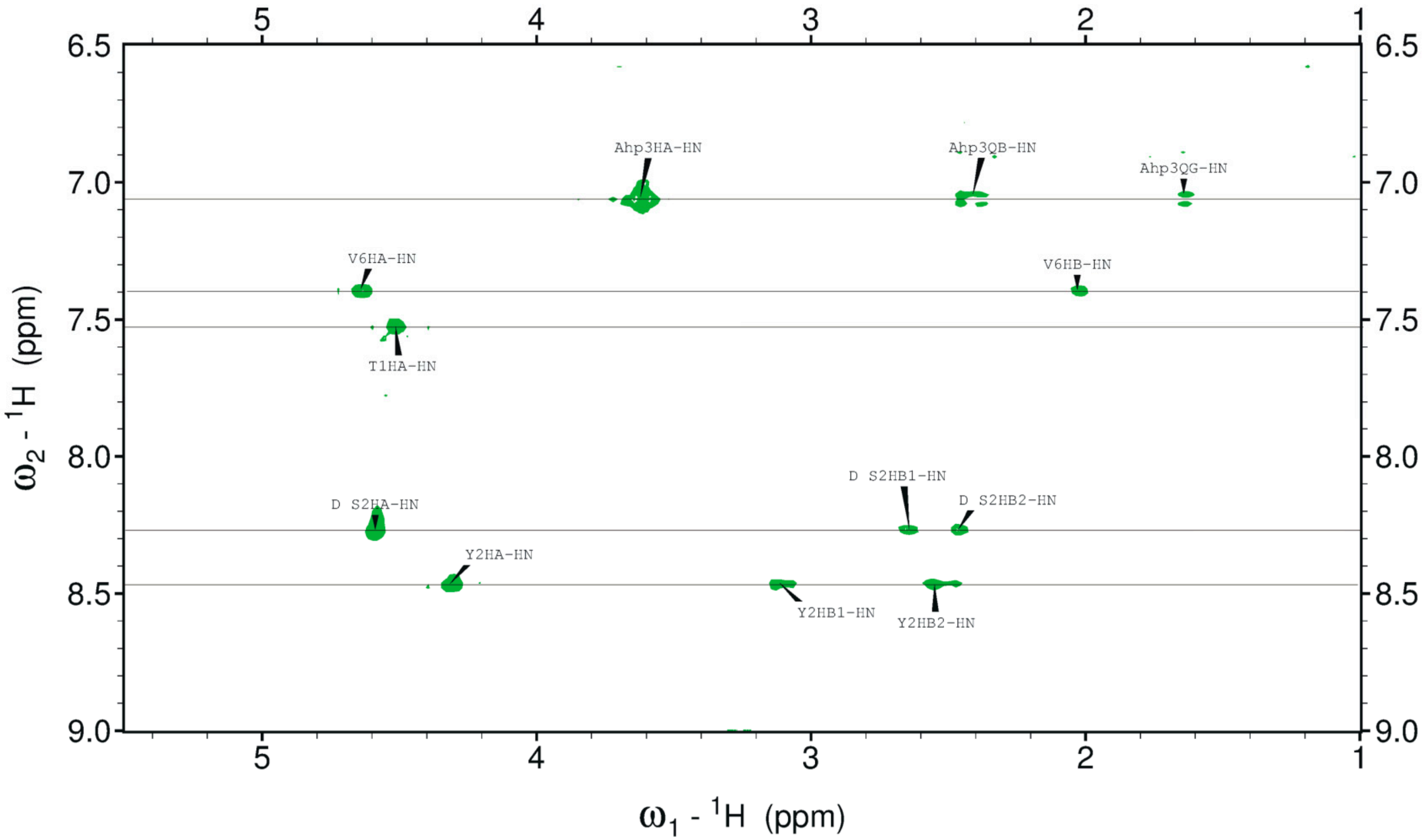

Figure S17b. Amino acid spin systems in the diagnostic region of the TOCSY spectrum of cyanopeptolin CP985.

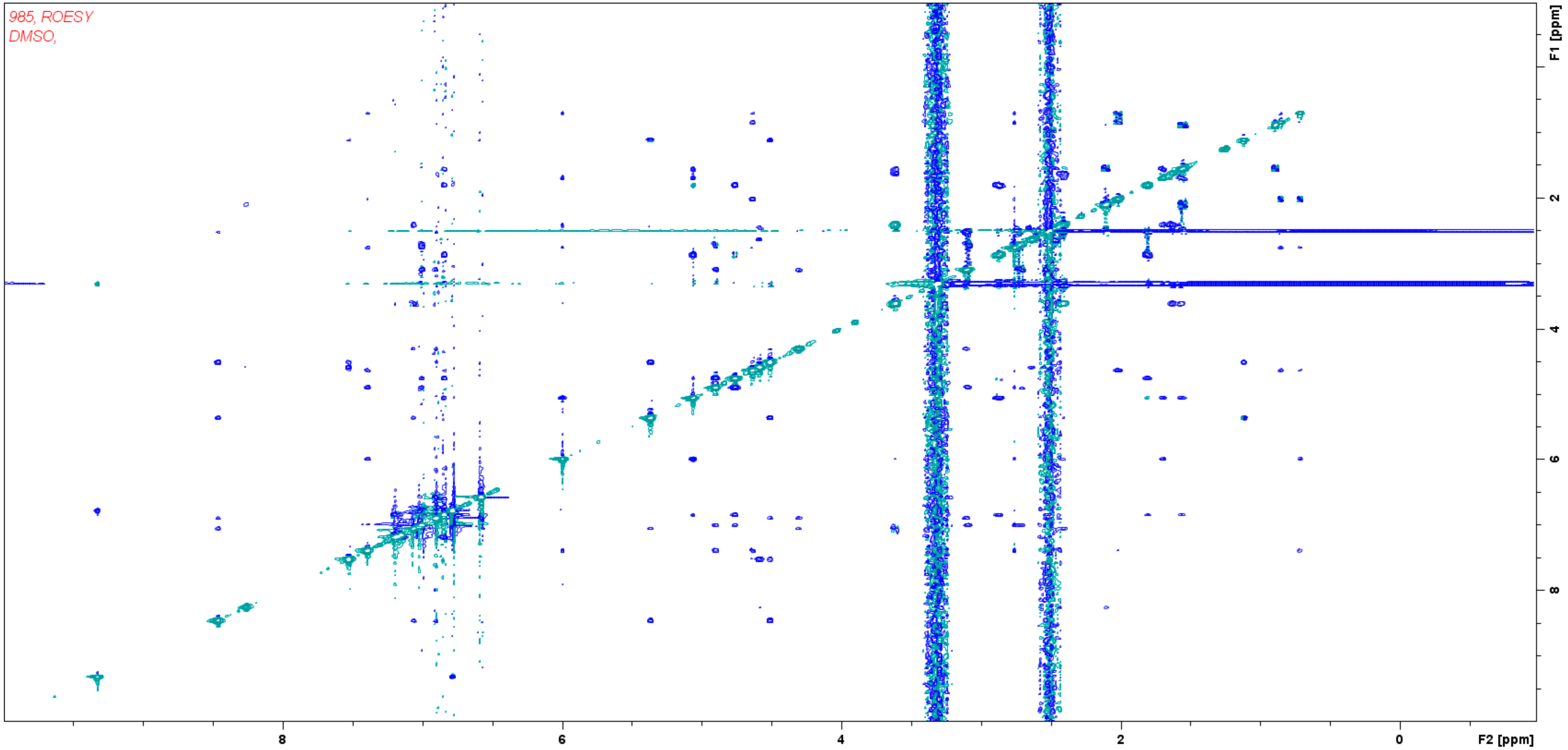

Figure S18a. ROESY Spectrum of cyanopeptolin CP985 in DMSO-d_6.

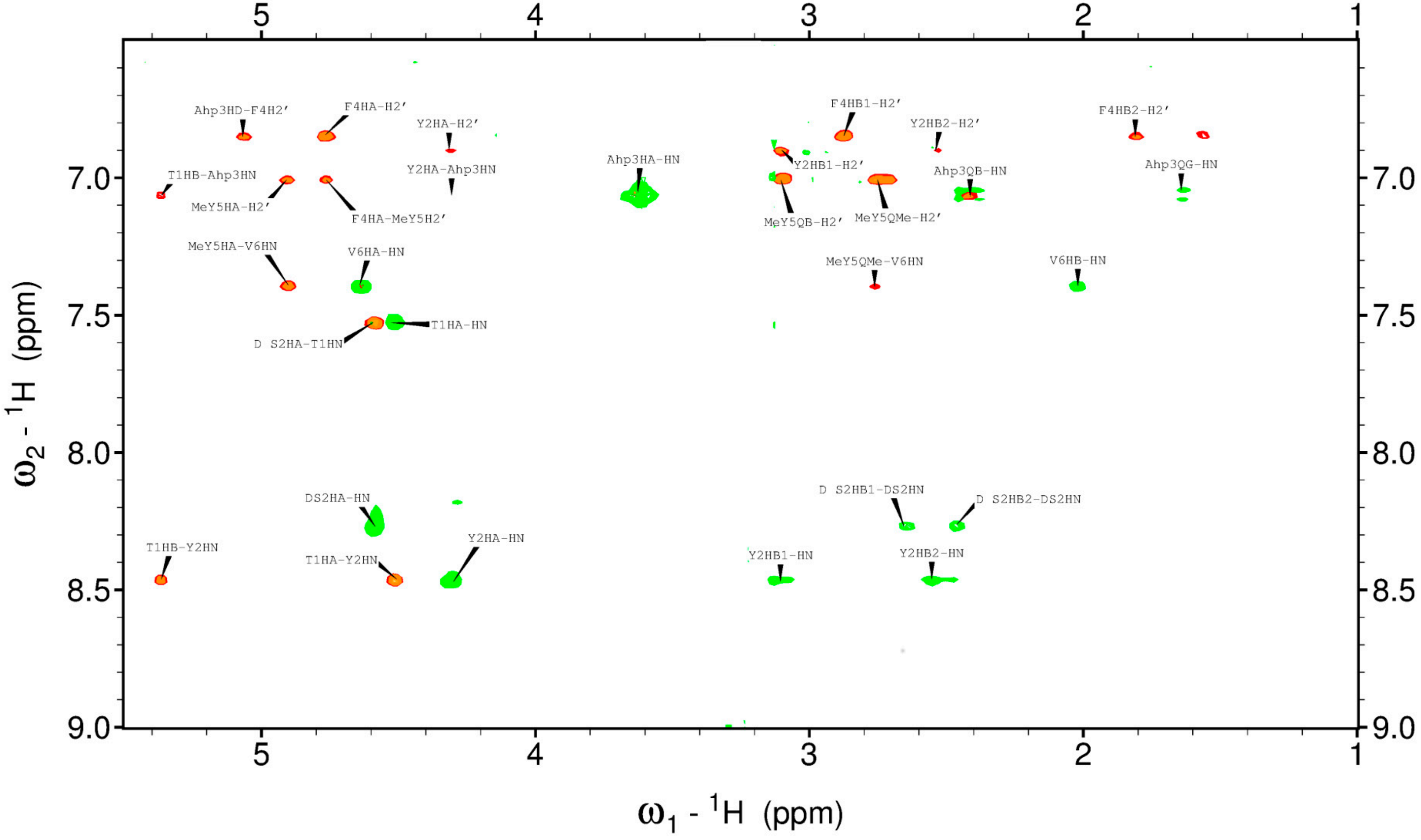

Figure S18b. Overlaid fragments of TOCSY (green) and ROESY (red) spectra of cyanopeptolin CP985.

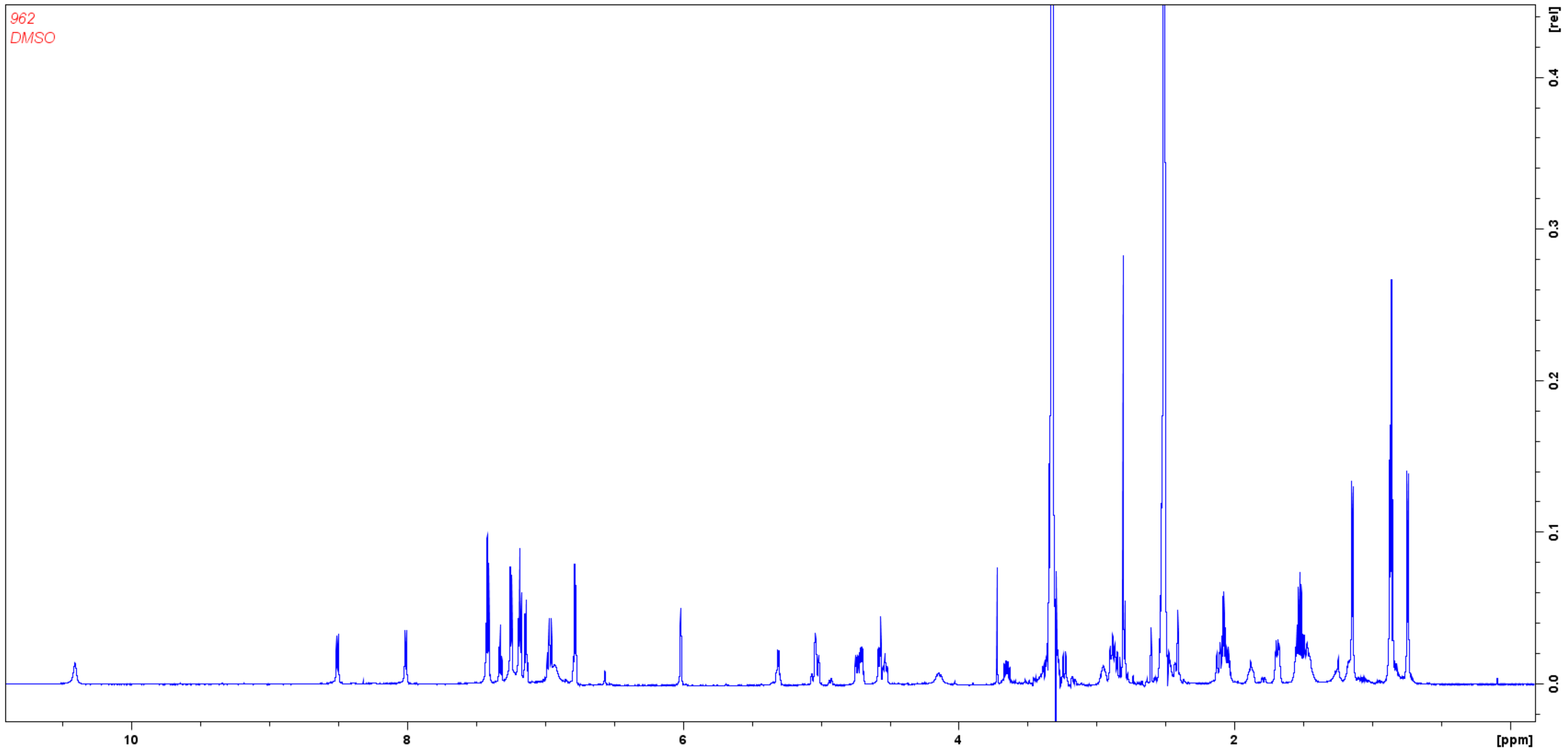

Figure S19. ^{1}H NMR Spectrum of cyanopeptolin CP962 in DMSO-d_6.

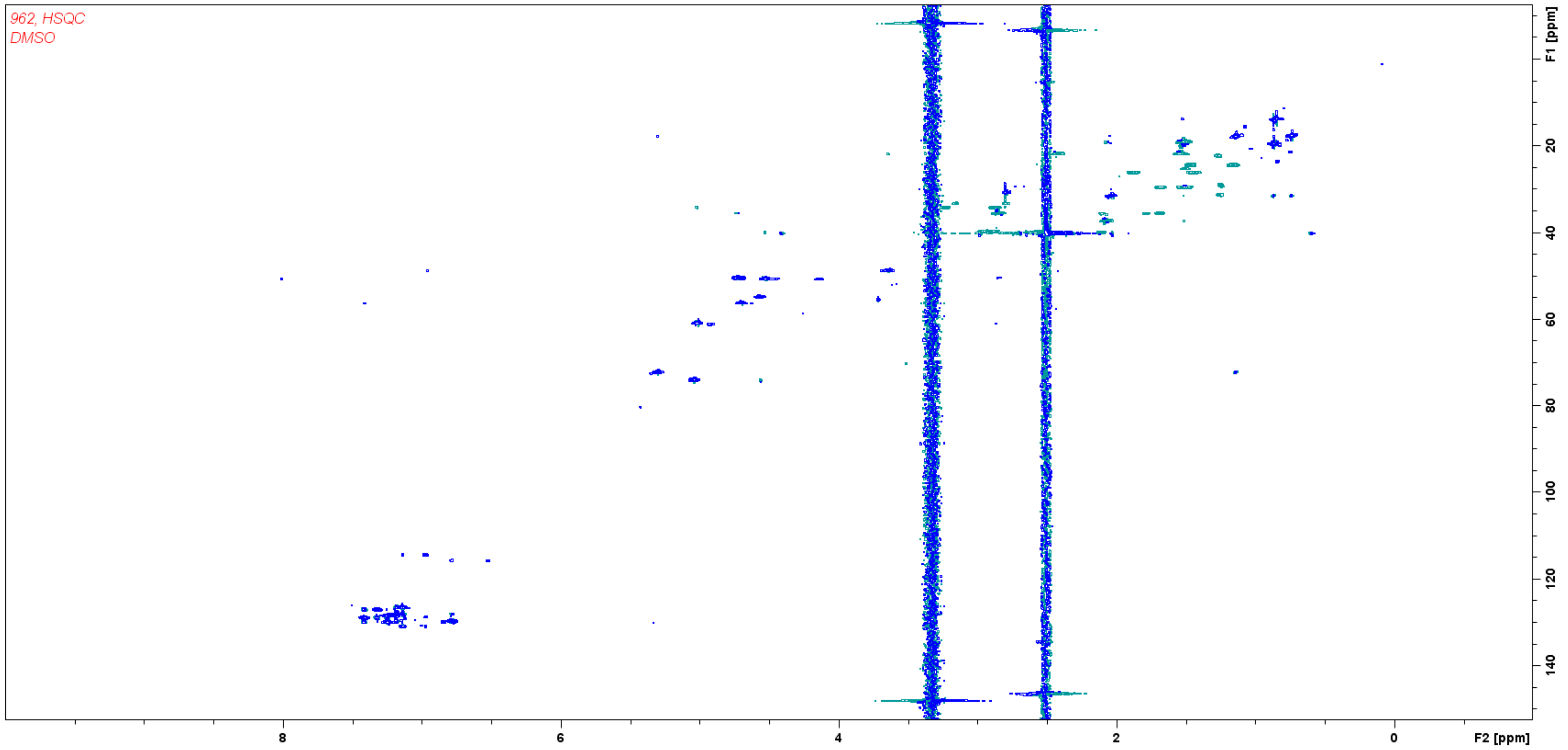

Figure S20. HSQC Spectrum of cyanopeptolin CP962 in DMSO-d6.

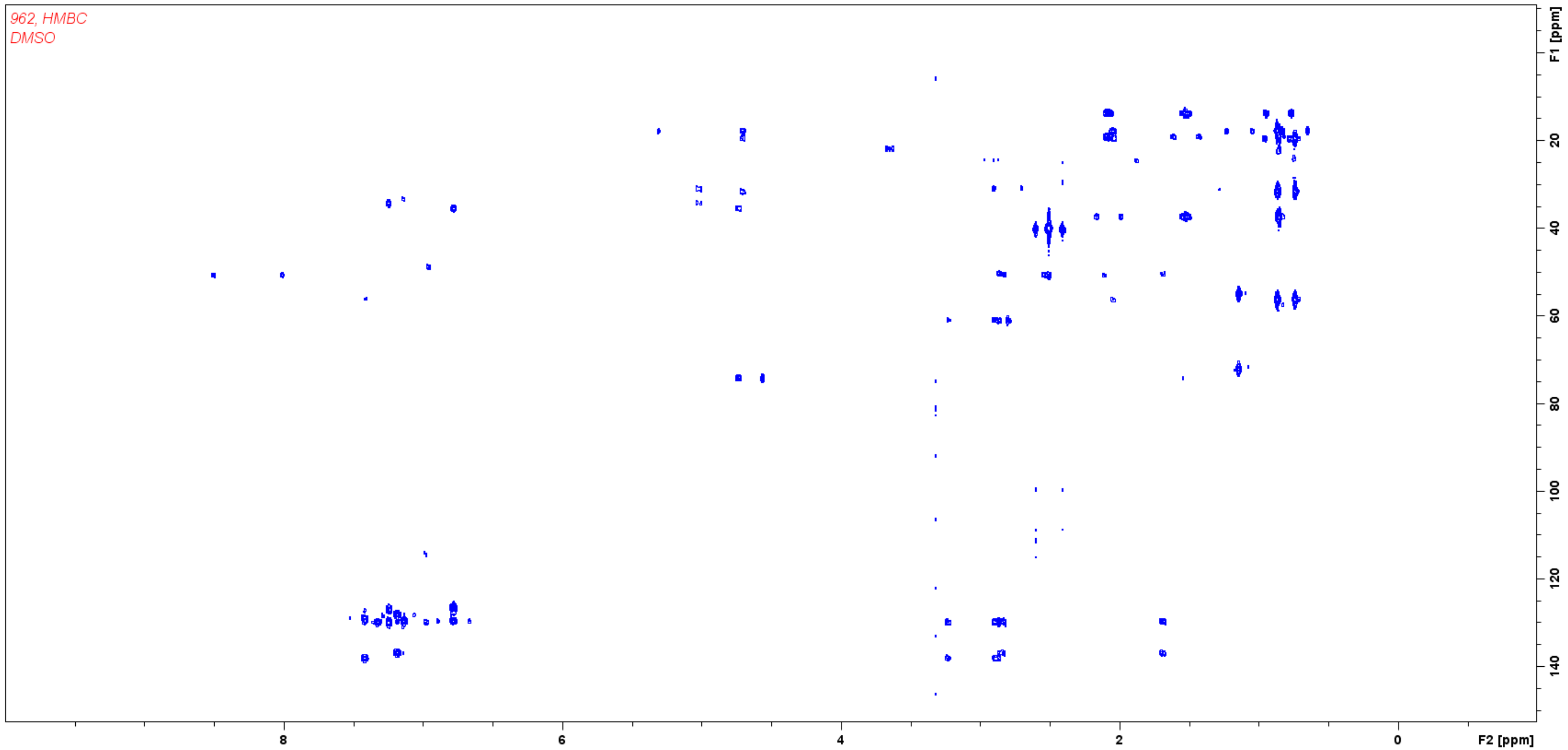

Figure S21a. HMBC Spectrum of cyanopeptolin CP962 in DMSO-d6.

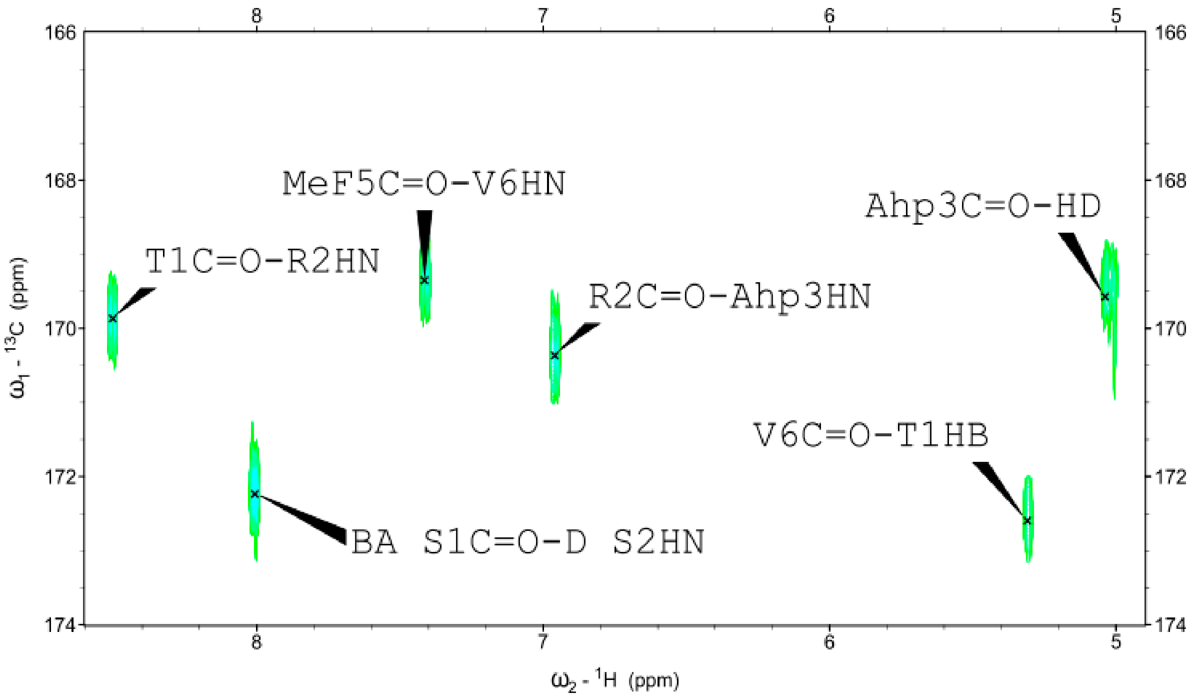

Figure S21b. Detailed NH – C=O region of the HMBC spectrum of cyanopeptolin CP962.

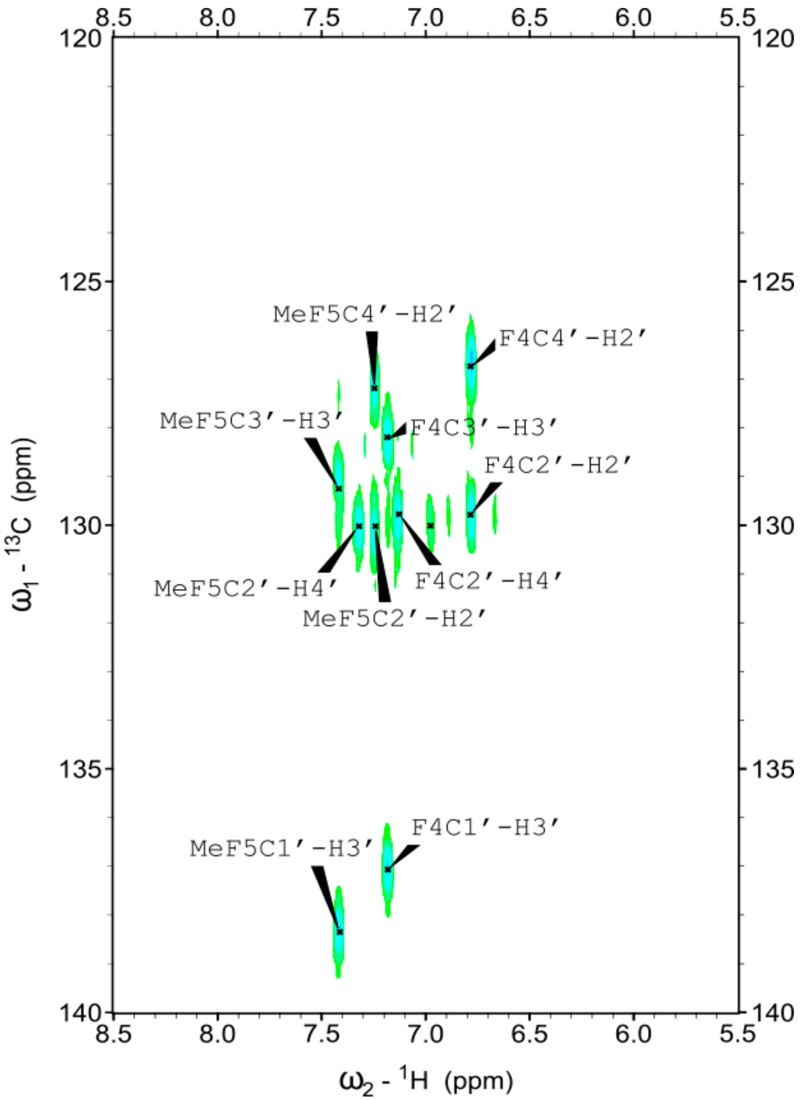

Figure S21c. Detailed aromatic region of the HMBC spectrum of cyanopeptolin CP962.

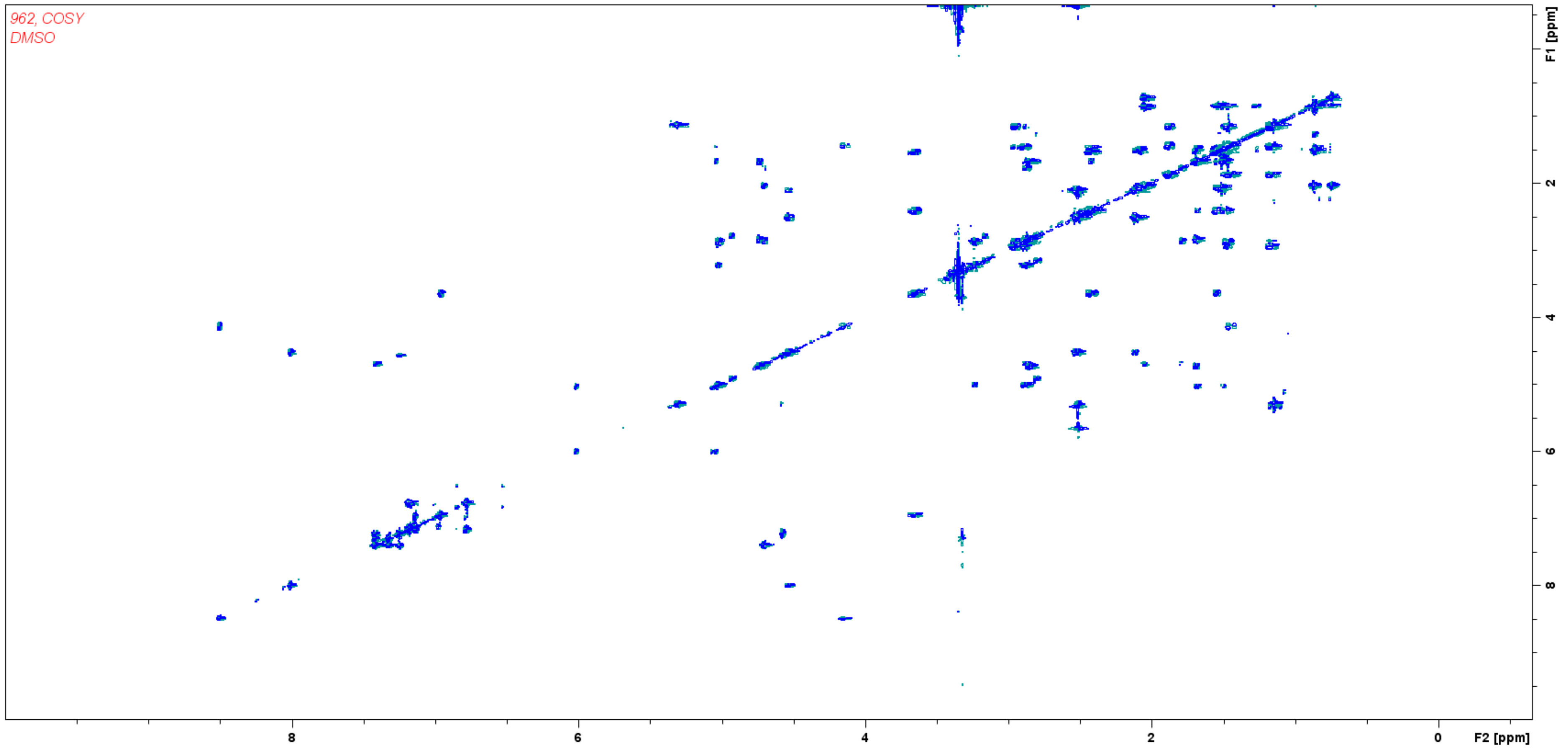

Figure S22. COSY Spectrum of cyanopeptolin CP962 in DMSO-d_6.

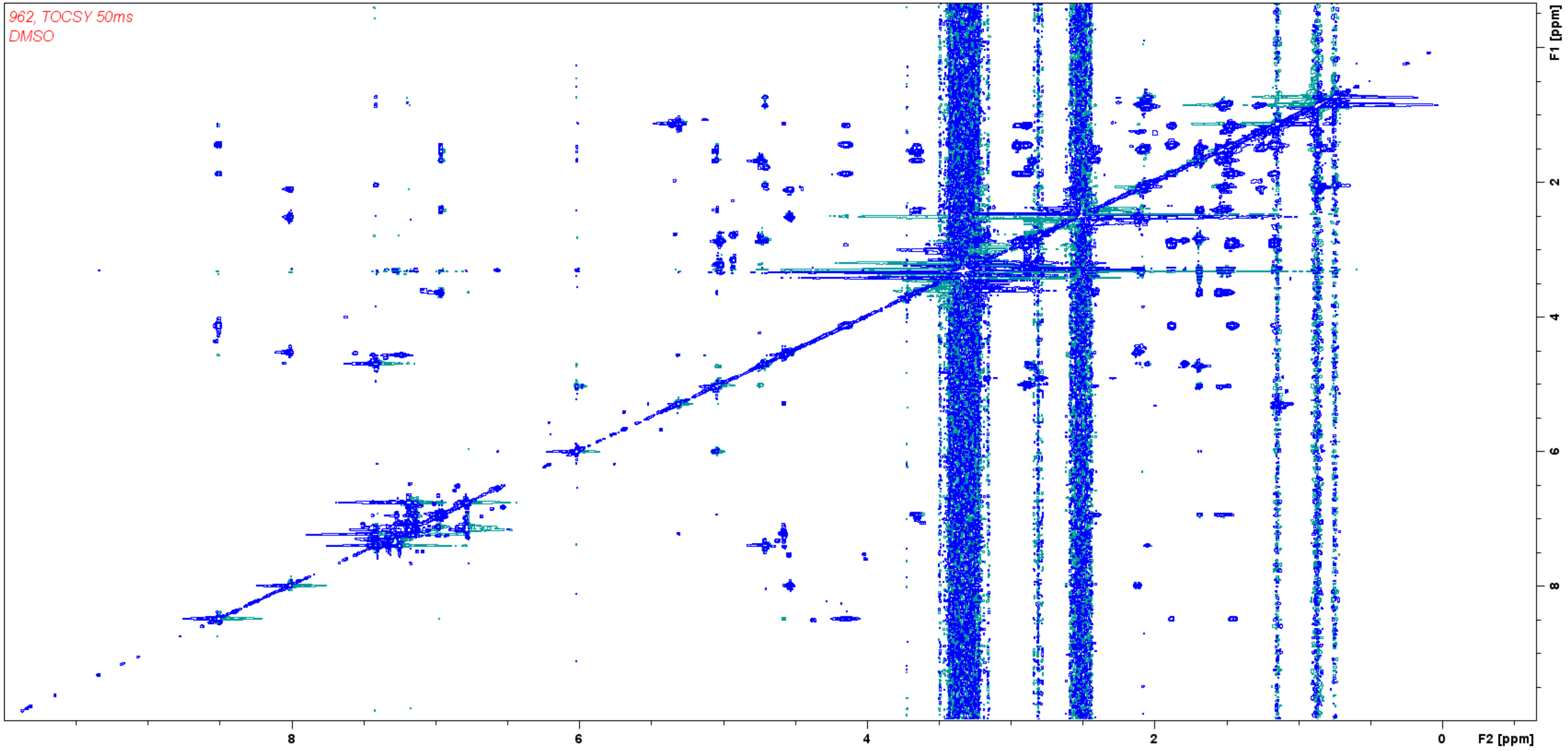

Figure S23a. TOCSY Spectrum of cyanopeptolin CP962 in DMSO-d6.

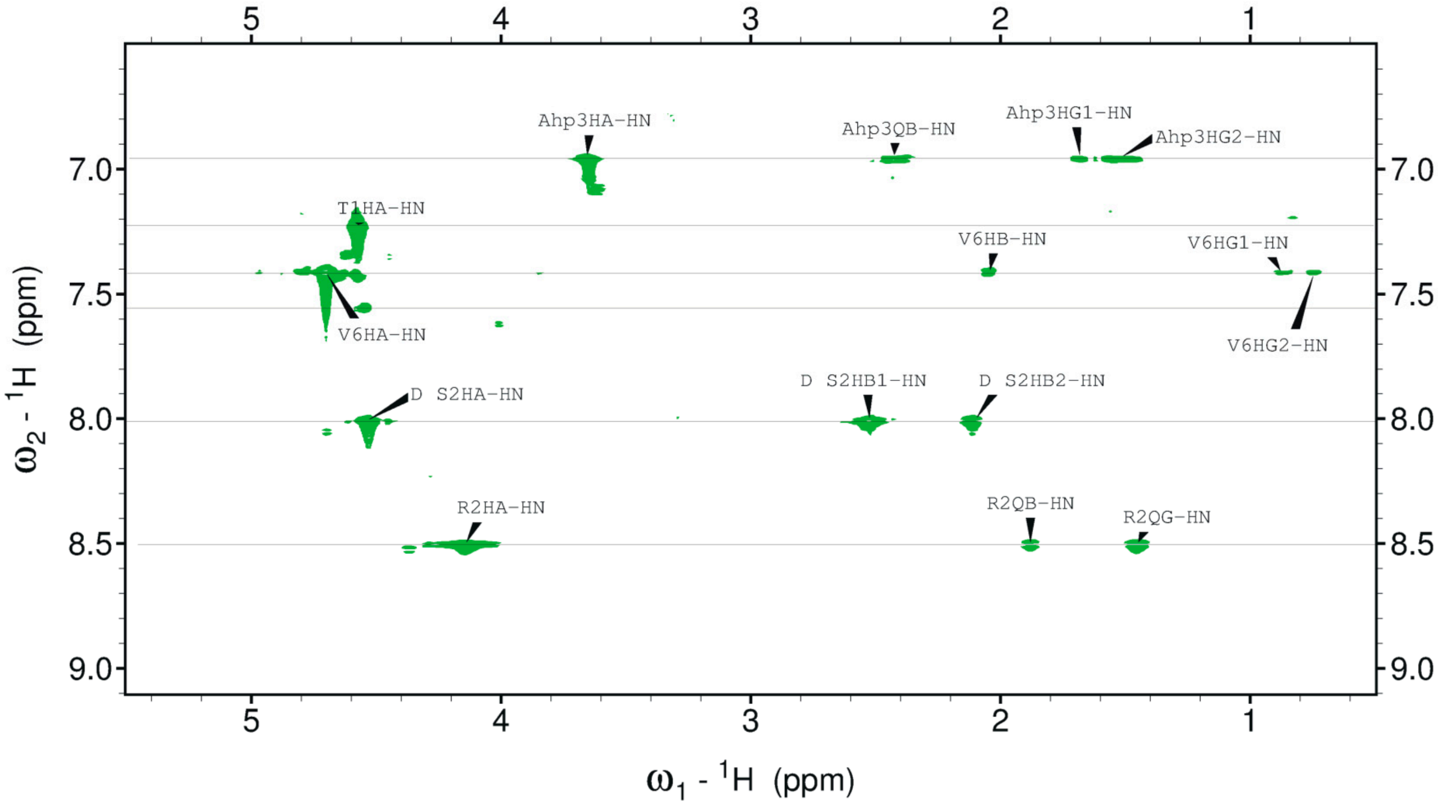

Figure S23b. Amino acid spin systems in the diagnostic region of the TOCSY spectrum of cyanopeptolin CP962.

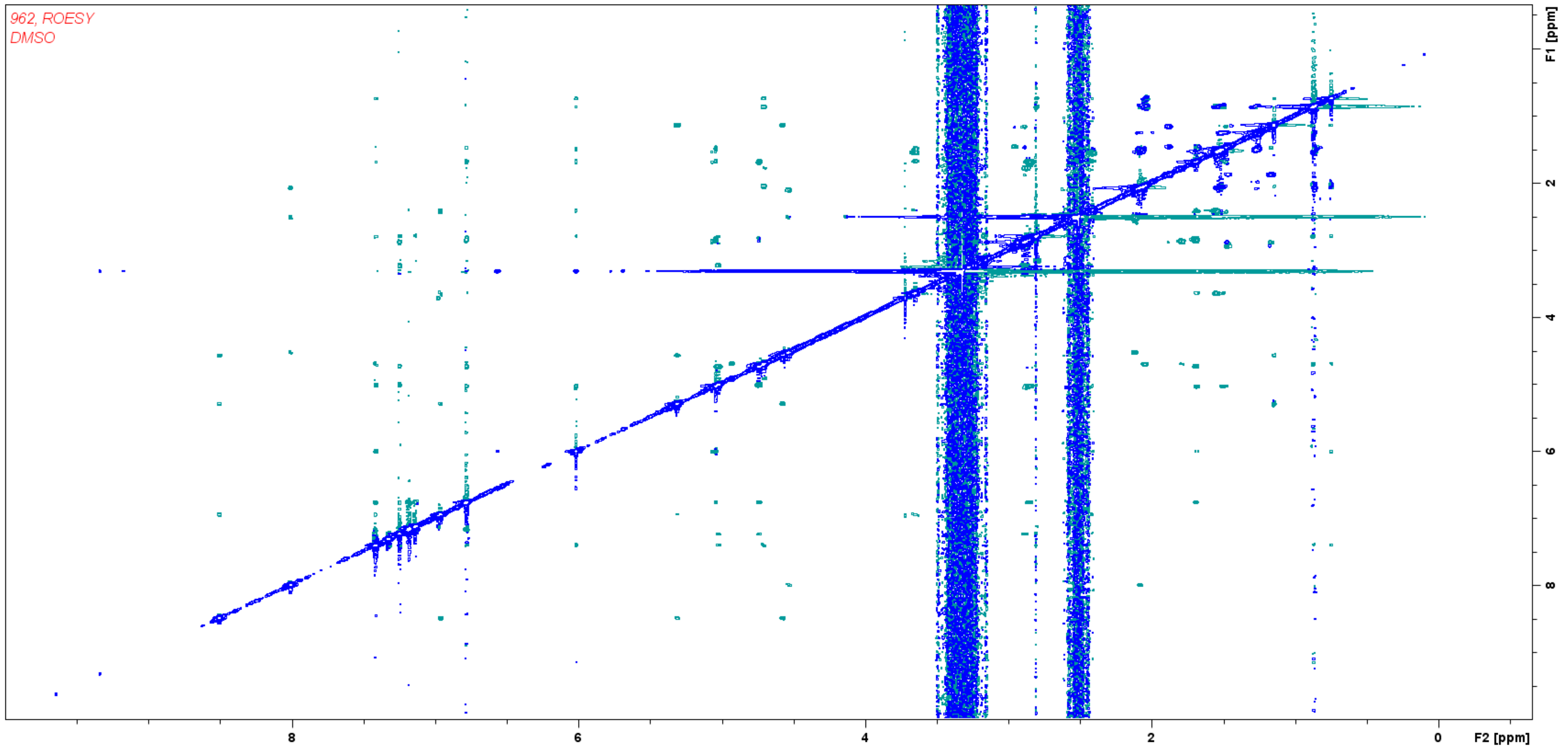

Figure S24a. ROESY Spectrum of cyanopeptolin CP962 in DMSO-d6.

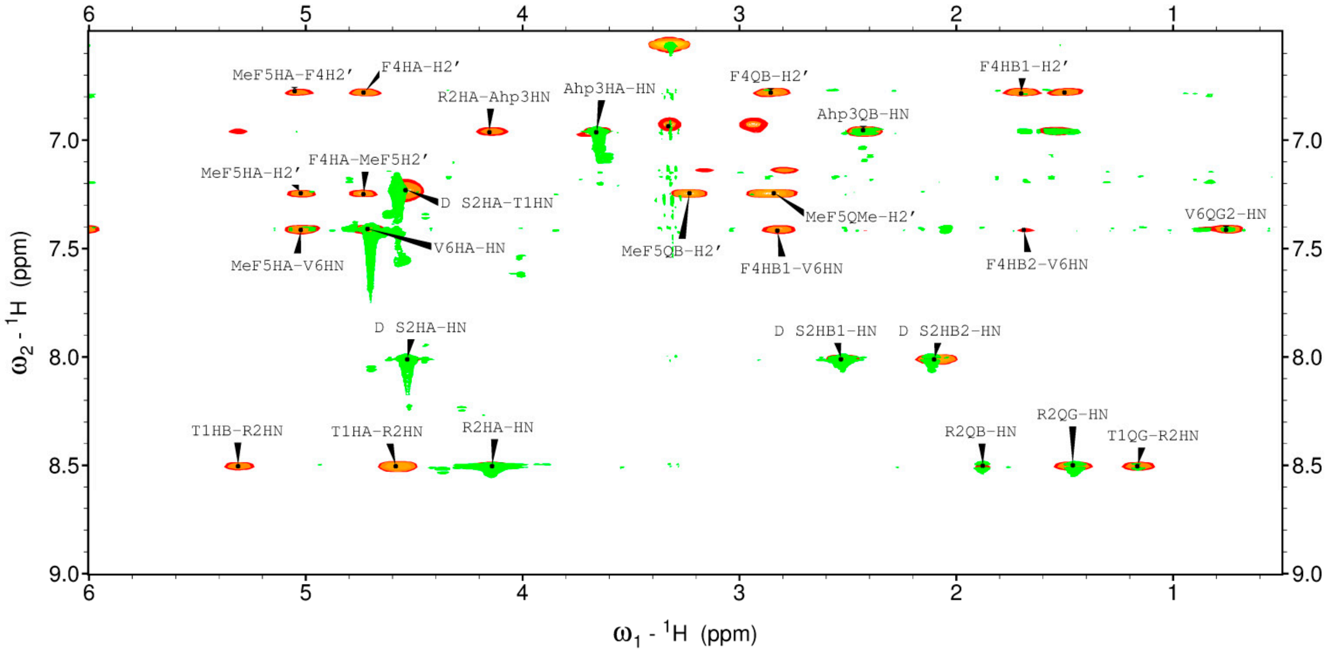

Figure S24b. Overlaid fragments of TOCSY (green) and ROESY (red) spectra of cyanopeptolin CP962.
